# Prediction of peak particle velocity using hybrid random forest approach

**DOI:** 10.1038/s41598-024-81218-z

**Published:** 2024-12-28

**Authors:** Yu Yan, Jiwei Guo, Shijie Bao, Honglu Fei

**Affiliations:** 1 Present Address: School of Civil Engineering, Liaoning Technical University, Fuxin, 123000 China; 2 Collaborative Innovation Center of Mine Major Disaster Prevention and Environmental Restoration, Fuxin, 123000 China; 3 Institue of Blasting Technology, Liaoning Technical University, Fuxin, 123000 China

**Keywords:** Burden, Blast-induced ground vibration, Peak particle velocity, Machine learning algorithms, Arithmetic optimization algorithm, Mineralogy, Civil engineering, Engineering

## Abstract

Blasting excavation is widely used in mining, tunneling and construction industries, but it leads to produce ground vibration which can seriously damage the urban communities. The peak particle velocity (PPV) is one of main indicators for determining the extent of ground vibration. Owing to the complexity of blasting process, there is controversy over which parameters will be considered as the inputs for empirical equations and machine learning (ML) algorithms. According to current researches, the burden has controversial impact on the blast-induced ground vibration. To judge whether the burden affects blast-induced ground vibration, the data of ground vibration considering burden have been recorded at the Wujiata coal mine. Correlation coefficient is used to analyze the relationship between variables, the correlation between the distance from blasting center to monitored point (*R*) and peak particle velocity (PPV) is greatest and the value of correlation coefficient is − 0.67. This study firstly summarizes the most common empirical equations, and a new empirical equation is established by dimension analysis. The new equation shows better performance of predicting PPV than most other empirical equations by regression analysis. Secondly, the machine learning is confirmed the applicability of predicting PPV. Based on the performance assessments, regression error characteristic curve and Uncertainty analysis in the first round of predicting PPV, the random forest (RF) and K-Nearest Neighbors (KNN) show better performance than other four machine learning algorithms. Then, in the second round, based on the artithmetic optimization algorithm (AOA), the optimized random forest (AOA-RF) model as the most accurate model compared with the optimized K-Nearest Neighbors (AOA-KNN) presented in the literature. Finally, the points of predicted PPV which have been informed of danger are marked based on Chinese safety regulations for blasting.

## Introduction

With the acceleration of urban modernization, the number of engineering projects (such as mining, tunnels, and highways) increases rapidly. Meanwhile, rock fragmentation is a critical task in tunneling and mining and relies on drilling and blasting for a predetermined purpose using explosives. Blasting excavation is regarded as one of the most economical methods in tunnelling and mining^1^. However, in blasting operation, only 20–30% of the energy produced by the explosive is accounted for by rock fragmentation, and the remaining energy is mostly converted into environmental issues, such as air-overpressure, ground vibrations, and flyrock^2^. Ground vibrations are the most concerning among these adverse effects due to the damage they could cause to the nearby communities (e.g., structures, underground spaces, and humans) if not adequately controlled.

To solve the problems of blast-induced vibration, researchers studied the peak particle velocity (PPV), which is the basis for most regulations and can be easily predicted. The most common methods for PPV prediction include empirical equations, machine learning, and numerical simulation methods. Based on the widely used empirical equations for predicting the surface vibration, most researchers^3^ considered the distance between the monitored point and the blast source, the maximum charge per delay, and geological conditions as the influential factors to predict PPV. The constants *K*, $\alpha$, $\beta$, and *n* are determined using the multiple regression analysis. In contrast to other empirical equations, Murmu et al.^4^ considered burden (denoted *B* and measured in m) is as an influential parameter. Several relevant empirical equations are presented in Table 1.^5,6^

**Table 1:** Empirical equations for predicting PPV.

References	Empirical equation	References	Empirical equation
Duvall	${\text{v}} = k\left( {\frac{R}{\sqrt Q }} \right)^{ - \alpha }$	Gupta	${\text{v}} = k \cdot \left( {\frac{R}{\sqrt Q }} \right)^{ - \alpha } e^{{ - \beta \left( {R/Q} \right)}}$
Langefors	${\text{v}} = k\left( {\frac{{\text{Q}}}{{\sqrt[3]{{R^{2} }}}}} \right)^{{\frac{\alpha }{2}}}$	Bilgin	${\text{v}} = k\left( {\frac{R}{\sqrt Q }} \right)^{ - \alpha } {\text{B}}^{\gamma }$
Ambraseys	${\text{v}} = k\left( {\frac{R}{{\sqrt[3]{Q}}}} \right)^{ - \alpha }$	Roy	${\text{v}} = {\text{n}} + k\left( {\frac{R}{\sqrt Q }} \right)^{ - 1}$
Murmu	${\text{v}} = k \cdot \left( {\frac{R}{{Q^{2/5} }}} \right)^{ - \alpha }$		

Where *v* is the peak particle velocity (in cm/s), *K* and $\alpha$ are empirical constants, *Q* is the maximum charge weight per delay (in kg), and *R* is the distance between blasting source and the monitored point (in m). The ratio *R/Q*^*n*^ is known as scaled distance. Number *n* is uniform and varies across studies.

Many researchers aimed to predict PPV by investigating the influences of various parameters on ground vibrations. These parameters can be divided into two categories^4^, namely, controllable and uncontrollable parameters. Controllable parameters are blast design parameters (e.g., ‘hole diameter’, ‘hole depth’, ‘burden’, ‘spacing’, ‘stemming’, ‘sub-drilling’, and ‘number of holes’) and explosive parameters (e.g., ‘explosive type’, ‘maximum charge per delay’, ‘total charge’, and ‘delay time’). Uncontrollable parameters include geotechnical and geo-mechanical parameters. Recent studies typically consider *R*, *Q,* and geological conditions as the major factors in predicting PPV. However, empirical equations yielded inaccurate estimations of PPV, leading to the consideration of additional factors affecting ground vibrations^7^. To predict blast-induced vibration accurately, Yan et al.^6^ summarized the influential parameters’ mechanisms that affect PPV prediction and reviewed the models which can predict the ground vibration caused by blasting. The study shows that free faces, charge structure, and charge parameters have effects on ground vibration, as rare studies on stemming and spacing did not offer sufficient evidence proving its impact on the prediction. Meanwhile, the influences of a burden on the ground vibrations were found to be controversial, some researches found that the burden has an impact on the peak particle velocity, while others believe that it does not.

**Fig. 1:**
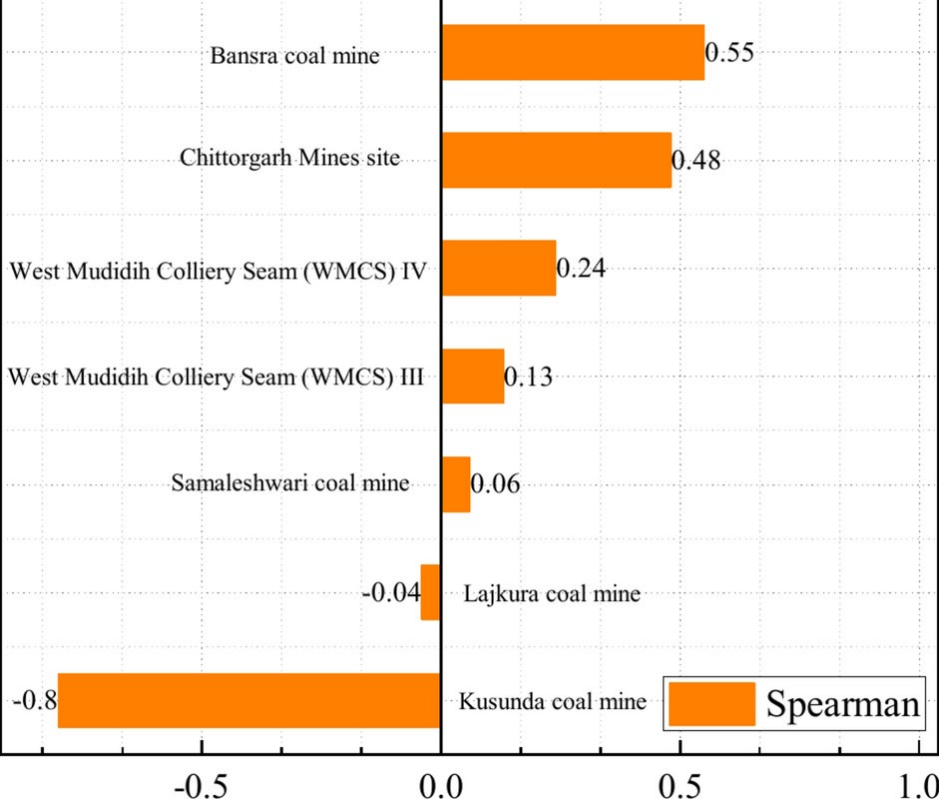
The correlation matrix between burden and PPV.

Burden, delineated as the minimum distance between the explosion center and the free face, exerts influence on the shape of the blasting crater, the extent of dispersion, and the fragmentation. Bergmann et al.^8^ first studied the effect of burden on the prediction of PPV by blasting measured results, and found that it was not obviously influence of burden on ground vibration. Based on the test results, Blair et al.^9,10^ indicated that the burden did not affect on the blast-induced ground vibration. In contrast, Liu et al. ^11^ and Uysal et al.^12^ measured the ground vibration with different lengths of burden, and concluded that burden was the influential parameter on the blast-induced ground vibration. Murmu^4^ collected 640 blast data to investigate whether it was possible to take into account the burden in the important parameters and to study the influence of burden on PPV. According to the collected data, the correlation matrix (Spearman) between burden and PPV is investigated. Figure 1 shows the correlation matrix in each recorded dataset, and indicates that a contentious association between burden and ground vibration. Consequently, empirical examination is warranted to elucidate the effects of burden on the prognostication of blast-induced ground vibration.

With the development of statistics and computer science, researchers started to employ ML to tackle complex engineering problems^13–15^. Coincidentally, due to the limited parameters considered by empirical equations and their low prediction accuracy, numerous new ML algorithms emerged and have been applied to practical engineering^16,17^. Several studies^18,19^ considered different parameters when designing artificial neural network (ANN) models, which displayed better performance than empirical equations. Apart from ANN, there are numerous algorithms adopted to predict the ground vibration, including, for example, support vector regression (SVR), classification and regression tree (CART), and adaptive neuro-fuzzy inference system (ANFIS)^20–22^. These algorithms’ performance demonstrates the superiority of ML techniques over empirical equations. Moreover, Faradonbeh et al.^23^ proposed a model for predicting PPV using the gene expression programming (GEP) algorithm. This algorithm creates mathematical expressions fitted to the training set data, generating an explicit expression for predicting PPV. The results show the GEP algorithm predicts PPV with good prediction accuracy. Zhou et al.^24^ developed RF model as a new model in predicting PPV, the accuracy level of the FS-RF model is quite 92.95% and 90.32% for the train and test stages, respectively. Table 2 summerized the ML algorithms which were adopted to predict PPV. Hosseini^25^ developed a black hole optimized long short-term memory (BH-LSTM) for predicting PPV with considering six parameters. The performance of the BH-LSTM model showed higher accuracy than other models, which can be used to predict the blast-induced ground vibration and ensure the safety of ubran communities. Fissha^26^ applied an optimised RVM models to predict ground vibration prediction by comparing 33 machine learning models, and found that the PSO optimized dual kernel based RVM model (PSO-DRVM) had the smallest residual in predicting PPV.

The researchers employed different empirical equations and machine learning models to predict PPV by different databases. Specially, Machine learning (ML)^27,28^ has become instrumental in engineering due to its ability to address the limitations of empirical equations, which often fall short when modeling complex phenomena with limited parameters. ML techniques, by contrast, can model non-linear relationships across multiple variables, leading to significantly higher prediction accuracy. However, the parametric study was not first determined to judge which parameter was influential before the ML models are trained. For example, the influence of burden, hole depth and hole diameter is not considered in most researches. Besides, the conventional machine and advanced machine are only compared in predicting PPV, without considering the selection of models with high predictive accuracy by comparing traditional ML models, followed by optimization to enhance prediction efficiency and accuracy.

**Table 2:** The summerized ML algorithms for predicting PPV.

Researchers		No. of parameters	ML algorithm	R^2^
Qiu^29^	HR, HD, B, *S*, Q, CL, R, BI, *E*, PR, Pv, VoD, DoE	13	(WOA-, GWO-, BO-)XGBoost	0.9757
Zhang^30^	Q, R, St, B, H, *S*, PF, HN	8	PSO-XGBoost	0.968
Nguyen^31^	N, Q, PF, St, B, R, *S*, H, Δt	9	XGBoost	0.952
Amiri^32^	Q, R	2	KNN;ANN	0.88
Armaghani^33^	Q, R	2	ANN	0.987
Ghoraba^34^	Q, R	2	ANFIS	0.952
Hasanipanah^35^	Q, R	2	CART	0.95
Khandelwal^36^	Q, R	2	SVM	0.96
Khandelwal^22^	Q, R	2	CART	0.92
Arzu^37^	Q, R, S*v*, *S*, B	5	ANFIS	1
Monjezi^38^	Q, R, *S*, HD	4	ANN	0.9493
Nguyen^39^	B, S, *f*, Q, R	5	(SpaSO-, SalSO-,MFO-)ELM	0.99
Monjezi^40^	Q, R, B/*S*, UCS, RW, HN	6	MLPNN	0.954
Nguyen^41^	B, S, *f*, PF, Q, R	6	(MRFO-, HGS-, AO-) SONIA	0.896
Yang^42^	B, S, St, Q, R	5	FFA-SVR	0.992
Zhou^24^	HD, PF, S, Q, R	5	FS-RF	0.903
Hosseini^25^	HN,B,H,HR,Q,R	6	BH-LSTM	0.9956
Fissha^26^	Q, R	2	PSO-DRVM	0.917

Based on the presented discussion, this study aims to investigate the effect of burden on predicting PPV and develop a data-driven ML approach for estimating blast-induced ground vibrations. A field study at Wujiata coal mine was conducted to investigate the prediction performance of several ML algorithms. Firstly, the correlations between PPV and input parameters are determined to investigate the parameters’ influence on ground vibration. Secondly, the performances of different ML models and empirical equations are studied. While many ML algorithms are available for predicting PPV, the problem of different conditions has not been addressed. Therefore, a mathematical model and ML models are developed to predict the PPV of surface affected by the burden of open-mine blasting. Performance assessment is utilized and the predictive models’ performances are compared for verification and calibration.

## Empirical equations for predicting PPV with considering burden

Controlling the ground vibration level is critical to ensure the safety of surrounding communities. In particular, PPV is considered an important criterion to define damage to the surrounding urban communities. Therefore, many researchers investigated the characteristics of the PPV attenuation rule and established empirical equations for accurate PPV prediction through experiments and theoretical analysis. This section establishes a predictive model which considers the burden.

Previous studies have shown that PPV is affected by many factors. However, the type of explosive, explosive density, and rock mass characteristics are similar in actual blasting. A project often requires repeated blasting in the same area. Considering the influence of the burden, PPV in a coal mine can be expressed as:

1 $${\text{v}} = \phi (Q,R,c,{\text{B}},\rho )$$

As noted before, *Q* stands for the maximum charge per delay, *R* stands for the distance between the blasting center and the monitored point, and *B* stands for the burden. Further, *c* denotes the phase velocity, and $\rho$ is the rock mass density. According to Buckingham’s Pi theorem, the influential parameters ${\text{Q}}$, ${\text{R}}$, and ${\text{c}}$ are independent and satisfy the following formulae. Let $\pi_{{\text{n}}}$ donate the dimensionless form of the dependent variables, $\pi_{{\text{n}}}$ can be expressed as:

2 $$\left\{ {\pi _{0} = \frac{{\text{v}}}{c},\pi _{1} = \frac{\rho }{{QR^{{ - 3}} }},\pi _{2} = \frac{{\text{B}}}{R}} \right..$$

Therefore, the equation for predicting PPV can be expressed as:

3 $$\frac{{\text{v}}}{c} = \phi \left( {\frac{{\text{B}}}{R},\frac{\rho }{{QR^{ - 3} }}} \right).$$

A new dimensionless parameter *π*_3_ is introduced by combining *π*_1_ and* π*_2_ as follows:

4 $$\pi_{3} { = }\left( {\pi_{1}^{1/3} } \right)^{{\beta_{1} }} \pi_{2}^{{\beta_{2} }} = \left( {\frac{{\sqrt[3]{\rho }R}}{{\sqrt[3]{Q}}}} \right)^{{\beta_{1} }} \left( {\frac{{\text{B}}}{R}} \right)^{{\beta_{2} }} .$$

Since PPV of ground vibration is typically predicted in similar situations, the parameters $\rho$ and *c* are assumed to be constant. According to Eq. (4), the relation between *v* and $\left( {\frac{R}{{\sqrt[3]{Q}}}} \right)^{{\beta _{1} }} \left( {\frac{B}{R}} \right)^{{\beta _{2} }}$ can be written as:

5 $$\ln v = \left[ {\alpha_{1} + \beta_{1} \ln \left( {\frac{{\sqrt[3]{Q}}}{R}} \right)} \right] + \left[ {\alpha_{2} + \beta_{2} \ln \left( {\frac{{\text{B}}}{R}} \right)} \right].$$

Let $\ln v = \ln \alpha _{1} + \beta _{1} \ln \left( {\frac{{\sqrt[3]{Q}}}{{\text{R}}}} \right) + \ln \alpha _{2} + \beta _{2} \ln \left( {\frac{{\text{B}}}{R}} \right)$, where $\ln K_{1} = \ln \alpha_{1}$ and $\ln K_{2} = \ln \alpha_{2}$. Then, Eq. (6) can be written as:

6 $${\text{v}} = K_{1} {\text{K}}_{2} \left( {\frac{{\sqrt[3]{Q}}}{R}} \right)^{{\beta_{1} }} \left( {\frac{{\text{B}}}{R}} \right)^{{\beta_{2} }} .$$

Since $K_{1}$ and $K_{2}$ are constants, $K_{1} K_{2}$ is transformed into $K$. Further, $\beta_{1}$ is transformed into $\alpha$, and $\beta_{2}$ is transformed into $\beta$, yielding the PPV prediction model:

7 $${\text{v}} = K\left( {\frac{{\sqrt[3]{Q}}}{R}} \right)^{\alpha } \left( {\frac{{\text{B}}}{R}} \right)^{\beta }$$

## Methodology

### Machine learning workflow

Machine learning is a technology in which computers imitate human learning abilities. Due to its ability to solve high-dimensional non-linear problems, various ML algorithms have been widely employed in many engineering applications, including tunneling and mining. For example, aiming to reduce the damage to urban communities, researchers^43–45^ developed regression models for PPV prediction to prevent ground vibration from causing disasters.

This research selects ridge regression (RR), lasso regression (LR), support vector regression (SVR), artificial neural network (ANN), k-nearest neighbours (KNN) and random forest (RF) to predict ground vibration. These algorithms are characterized by great prediction and classification performance in different civil engineering issues^46,47^. The algorithms are briefly introduced in section “Overview of machine learning algorithms”.

The ML workflow shown in Fig. 1 is applied to develop and train a model for accurate PPV prediction (and, consequently, ground vibration prediction). The first step of this workflow is sampling. The data samples that include the input and output parameters are collected. To improve the regression model accuracy, it is necessary to analyze the dataset characteristics and select important features based on input parameters. The correlations between input parameters and PPV are quantified using the Pearson correlation coefficient. Let *x* and *y* denote two variables. Pearson correlation coefficient between *x* and *y* is calculated as:

8 $${\text{P}}_{xy} = \frac{Cov(x,y)}{{\sigma_{x} \cdot \sigma_{y} }} = \frac{{\sum\nolimits_{i = 1}^{n} {({\text{x}}_{i} - E(x))(y_{i} - E(y))} }}{{\sqrt {\sum\nolimits_{i = 1}^{n} {\left( {x_{i} - E\left( x \right)} \right)^{2} } } \cdot \sqrt {\sum\nolimits_{i = 1}^{n} {\left( {y_{i} - E\left( y \right)} \right)^{2} } } }}$$

where ${\text{Cov}}\left( {x,y} \right)$ is the population covariance matrix, $\sigma_{x}$ and $\sigma_{{\text{y}}}$ are the standard deviations of *x* and *y*, and $E\left( x \right)$ and $E\left( {\text{y}} \right)$ denote the average *x* and *y* values. Here, $x_{{\text{i}}}$ are independent variable values, whereas $y_{{\text{i}}}$ are dependent variable values.

**Fig. 2:**
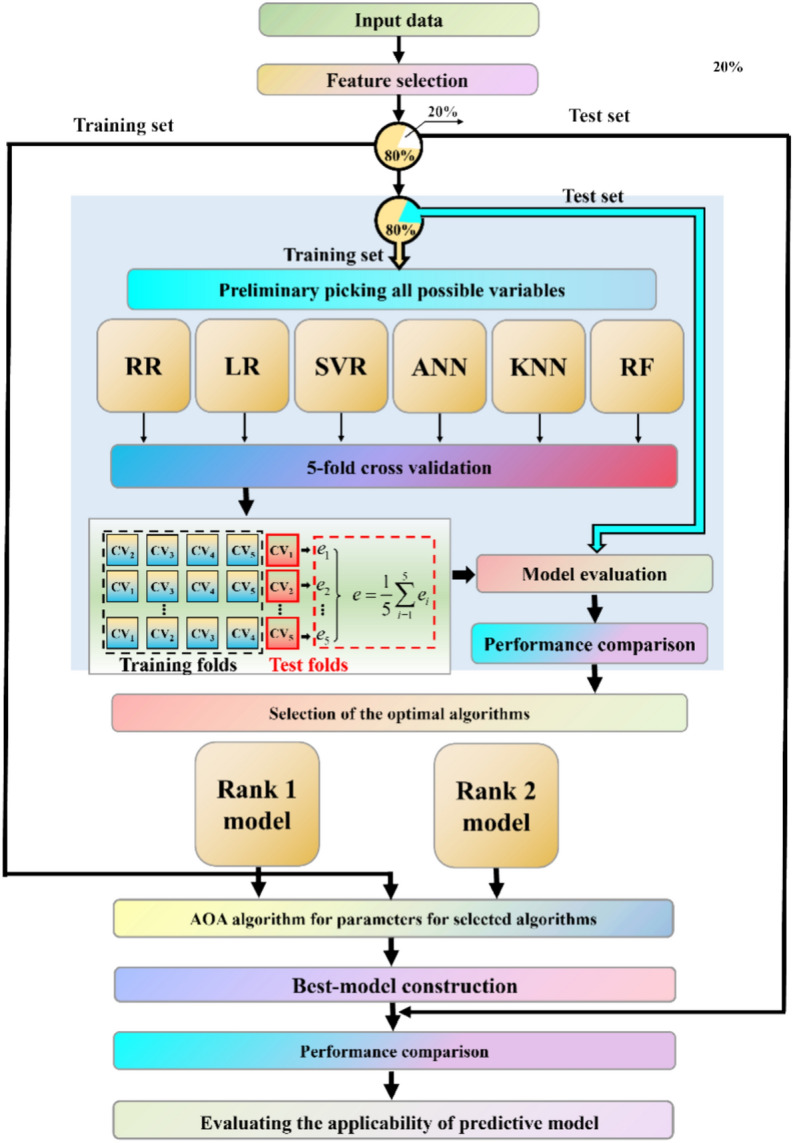
Machine learning (ML) workflow utilized in this study.

Feature selection is followed by the ML algorithms’ training. The training set contains 80% of data (64 data samples) sampled from the dataset, and the remaining 20% (16 data samples) forms the testing set. Of the training dataset, 55 samples (80% of the training set) are used for *k*-fold cross-validation of the six algorithms. *k*-fold cross-validation divides the original data into *k* groups. Then, *k* − 1 groups are used as a training set, and the remaining group serves as a test set to evaluate the algorithms. Such a process is repeated *k* times, each time using a different data group as a test set. This process enables evaluating the algorithms’ prediction performance while reducing overfitting. Therefore, this study uses fivefold cross-validation to improve the algorithms’ robustness. Upon cross-validation, each algorithm’s performance is evaluated using the remaining 9 data points (20% of the training set). This procedure enables identifying two algorithms with the highest accuracy, which is then selected and used for predicting PPV in the next round. These algorithms are denoted as ‘The best model’. Next, the algorithms’ hyper-parameters are optimized through an arithmetic optimization algorithm (AOA) and judging the models’ performance on the training set. Finally, the optimal model (i.e., the model with the highest accuracy) is selected to predict PPV and evaluate the influence range of PPV on urban communities. The detailed workflow is illustrated in Fig. 2.

### Overview of machine learning algorithms

#### Ridge regression (RR) and Lasso regression (LR)

Ridge regression, a regularized version of linear regression, is an improved least square method that mitigates the multicollinearity problem^48^. Unlike the least square method, ridge regression yields a biased estimation that may reduce the accuracy and disregard part of the information for the sake of obtaining a practical regression method. Let ${\text{D = }}\left\{ {\left( {x^{1} ,y^{1} } \right), \ldots ,\left( {x^{{\text{i}}} ,y^{i} } \right), \ldots ,\left( {x^{n} ,y^{n} } \right)} \right\}$, ${\text{x}}^{i} = \left\{ {x_{1}^{i} , \cdots ,x_{j}^{i} , \cdots ,x_{m}^{i} } \right\}$, and ${\text{y}}^{i} \in R$, where *m* represents the sample dimension and *n* denotes the number of samples. Further, let $\theta$ denote a vector of weight coefficients. Linear regression fits function ${\text{f}}_{\theta } \left( {x^{i} } \right) = \theta_{0} + \theta_{1} x_{1}^{i} + \theta_{2} x_{2}^{i} + \cdots + \theta_{m} x_{m}^{i}$ to dataset *D* by minimizing the cost function *J*(*θ*):

9 $${\text{J}}\left( \theta \right) = \frac{1}{n}\sum\limits_{i = 1}^{n} {\left( {f_{\theta } \left( {x^{{\text{i}}} } \right) - y^{i} } \right)^{2} } .$$

To prevent the linear regression from overfitting the data during the cost function minimization, RR and LR modify the cost function by adding a regularization item. The ${\text{l}}_{1}$ norm and ${\text{l}}_{2}$ norm of $\theta$ are utilized as regularization items, promoting the model generalizability and solving the problem of linear regression irreversibility. RR adopts the *l*_2_ norm, and its cost function is:

10 $${\text{J}}\left( \theta \right) = \frac{1}{n}\sum\limits_{i = 1}^{n} {\left( {f_{\theta } \left( {x^{{\text{i}}} } \right) - y^{i} } \right)^{2} } + \lambda \left\| \theta \right\|_{{^{2} }}^{2}$$

Lasso regression stands for Least Absolute Shrinkage and Selection Operator Regression^49^. Similar to RR, Lasso regression is also linear regression, but it adds the *l*_1_ norm to the cost function, i.e.:

11 $${\text{J}}\left( \theta \right) = \frac{1}{n}\sum\limits_{i = 1}^{n} {\left( {f_{\theta } \left( {x^{{\text{i}}} } \right) - y^{i} } \right)^{2} } + \lambda \left\| \theta \right\|_{1}$$

LR can eliminate the less-effective features by setting their corresponding weights to 0, deriving a sparse solution. Therefore, dimensionality reduction (feature selection) is realized during model training. These two algorithms not only ensure the error minimization by determining the best fitting function but also simplify the parameters and increase the model’s generalization ability.

#### Support vector regression (SVR)

**Fig. 3:**
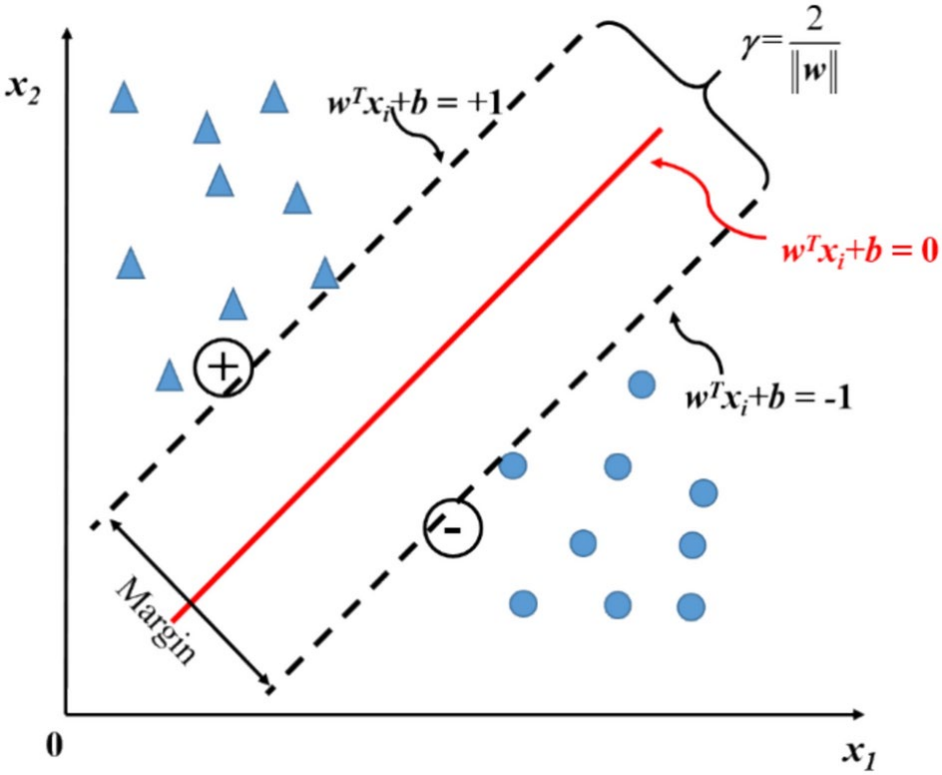
Support vectors and the margin.

Support vector machine (SVM) was initially proposed to solve classification and regression problems^50^. Support vector regression (SVR) uses similar principles as SVM but for regression problems. The SVM principle can be described as follows. The method aims to find an optimal hyperplane that separates the data into different categories, and such a hyperplane is the one having a maximum margin. The linear SVM model is shown in Fig. 3.

In classification problems, SVM does not calculate the loss when a sample point is far from the hyperplane. Similarly, for the regression problem, the loss is not calculated when the measured value is sufficiently distant from the predicted value. Their purpose is the same: to improve the model’s generalization ability. Assuming the database $XY = \left\{ {\left( {x,y} \right)\left| {\left( {x_{1} ,y_{1} } \right)} \right., \ldots ,\left( {x_{n} ,y_{n} } \right)} \right\}$ is the training sample, the SVR regression model between output $y_{k}$ and input $x_{k}$ can be expressed as:

12 $$y_{k} = f\left( {x_{k} } \right) = \left( {w,x_{k} } \right) + b$$

where *w* is the weight vector, *b* is the bias term. When $\left| {y_{k} - f\left( {x_{k} } \right)} \right| \le \varepsilon$ and the $\varepsilon$-intensive loss function is defined as $l_{\varepsilon }$, there will be no penalty in the optimization process for *w* and *b.* $l_{\varepsilon }$ can be expressed as:

13 $$l_{\varepsilon } = \left| {y - f\left( x \right)} \right|_{\varepsilon } = \max \left\{ {0,\left| {y - f\left( x \right)} \right| - \varepsilon } \right\}$$

The SVR model’s objective is to find the optimal hyperplane (or decision boundary), and solves a constrained optimization problem where the objective is to minimize a trade-off between model complexity, represented by the regularization parameter *C*, and the amount of deviation larger than ε allowed. The optimization problem in SVR can be formulated as:

14 $$\mathop {\min }\limits_{{w,b,\xi_{k} ,\xi_{k}^{*} }} \frac{1}{2}\left\| w \right\|^{2} + C\sum\limits_{k = 1}^{n} {\left( {\xi_{k} + \xi_{k}^{*} } \right)}$$

15 $${\text{subject}}\;{\text{to}}\left\{ {\begin{array}{*{20}l} {y_{k} - \left( {w,x_{k} } \right) - b \le \varepsilon + \xi _{k} } \hfill \\ {\left( {w,x_{k} } \right) + b - y_{k} \le \varepsilon + \xi _{k} } \hfill \\ {\xi _{k} ,\xi _{k}^{*} \ge 0} \hfill \\ \end{array} } \right.$$

where $\xi_{k} ,\xi_{k}^{*}$ are slack variables for data points outside the $\varepsilon$-insensitive zone. Therefore, selecting the appropriate value for these two parameters is critical. Generally, the smaller C values allow ignoring points close to the boundary and increase the margin. In contrast, large C values enable accurate regression of training points and can yield higher performance of SVR models in solving engineering problems. To optimize the convex quadratic program in Eqs. (14) and (15), a dual set of positive Lagrange multiplier variables is introduced. Only a subset of the training data, known as support vectors, contributes to the final model. These are the data points that lie on or outside the $\varepsilon$-margin and affect the positioning of the regression hyperplane. Points within the $\varepsilon$-margin have no effect on the solution, thereby enhancing the robustness of the SVR model. Once the SVR model is trained, the prediction for a new data point *x* is given by:

16 $$f\left( x \right) = \sum\limits_{k = 1}^{n} {\left( {\alpha_{k} - \alpha_{k}^{*} } \right)K\left( {x_{k} ,x} \right)} + b$$

where $\alpha_{k}$ and $\alpha_{{_{k} }}^{*}$ are the Lagrange multipliers from the optimization step, $K\left( {x_{k} ,x} \right)$ is the kernel function that maps the data into the high-dimensional space. The kernel function reduces the dimensional complexity of the calculations by replacing the inner product operation in high-dimensional feature space. Additionally, the kernel function directs the SVR models’ predictions.

#### ANN (Artificial neural network)

Artificial neural networks have been a research hotspot in the field of artificial intelligence since the 1980s. It is an algorithmic mathematical model that simulates the behavior characteristics of animal brains and processes distributed parallel information, and it can be used to learn and calculate functions with unknown analytical relationships between input and output. Artificial neural networks belong to the category of artificial intelligence, which reflects the operation mode of the human brain^51^. Like the human brain, an artificial neural network is composed of a series of interconnected neurons, which are arranged in layers.

**Fig. 4:**
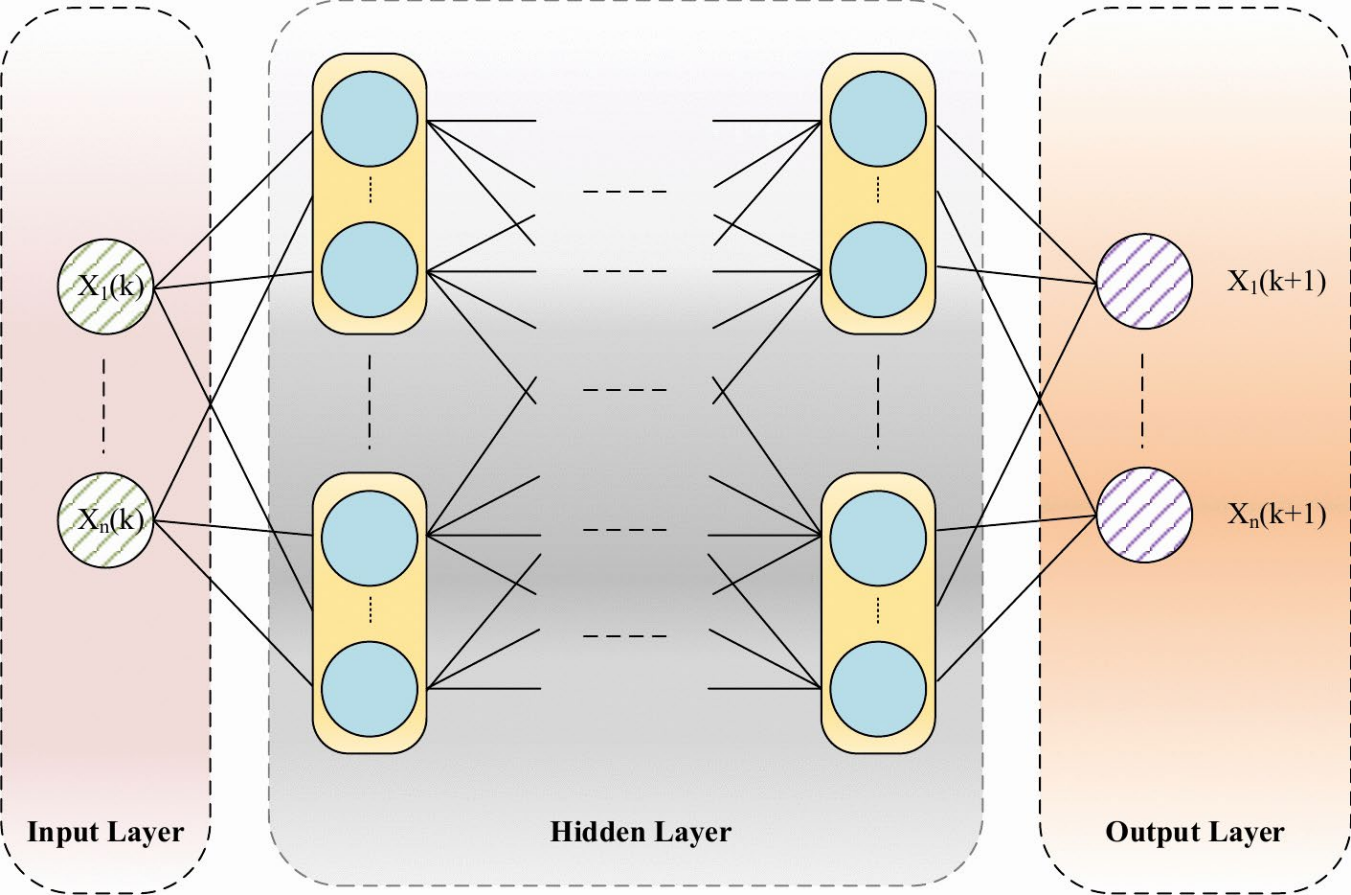
General representation of artificial neural network architecture.

An artificial neural network realizes the analysis and prediction of unknown functions by continuously adjusting the connection weights between neurons and training the complex relationship between input and output (Fig. 4). Its operation is similar to the human brain neuron network, in which each neuron represents a specific output function and processes the input signal through the activation function. It is a highly interconnected structure, which is composed of many simple processing elements (called neurons) and can perform large-scale parallel computing of data processing and knowledge representation. A neural network is first trained by processing a large number of input patterns and corresponding outputs. After proper training and prediction of the output mode, the neural network can identify the similarity of the new input mode. Applying Eqs. (17)–(18) to specific network configurations (i.e., the number of input, output, hidden layers, and nodes), a specific mathematical relationship between model input and output can be obtained, namely, it is a function of many unknown model parameters (i.e., connection weights and biases).

The input to the processing unit is:

17 $$I = \sum\limits_{i = 0}^{n - 1} {w_{i} } x_{i}$$

The output of the processing unit is:

18 $$y = f\left( {\sum\limits_{i = 0}^{n - 1} {w_{i} } x_{i} - \theta } \right)$$

where $x_{i}$ represents the input variables, $w_{i}$ represents the neuron connection weight. $f$ represents the activation function or the action function. $\theta$ represent the threshold of the hidden layer neural nodes.

#### K-nearest neighbors (KNN)

The K-Nearest Neighbors (KNN) algorithm, a versatile technique applicable to both regression and classification, hinges on the principle of ‘feature similarity’ for predicting values of new data points. By assigning a value based on the resemblance to training set points, KNN leverages proximity in the attribute space to infer similarities in the output space. Assuming a input point x, *K* identifies the K-nearest neighbors based on the chosen distance metric. Then, it computes the average of the target values (for regression) of these K neighbors:

19 $$\hat{y} = \frac{1}{k}\sum\limits_{i = 1}^{k} {y_{i} }$$

where $y_{i}$ is the target value of the i-th nearest neighbor, and $\hat{y}$ is the predicted value for *x*.The algorithm’s simplicity is reflected in its procedural steps: calculating distances between the new point and each training point, selecting the closest k data points, and determining the final prediction as the average of these selected data points. An inherent advantage of KNN lies in its resilience to noise, making it particularly adept at handling relatively large datasets. In essence, KNN offers a robust and straightforward approach to predictive modeling, catering to scenarios where feature relationships may be intricate or non-linear.

#### Random forest (RF)

Random forest (RF) is an ensemble method that utilizes a collection of decision trees, usually trained by bagging. RF was proposed in 1998 and can be used for both regression and classification^52^. It starts by employing the bootstrap method to extract *m* samples from the training set. Overall, *n* learning sample sets are formed through *n*-sampling. Each learning sample set then serves to train one decision tree, and each decision tree is independent of others in RF. For each decision tree, the attribute associated with the highest information gain is selected as the root node, effectively splitting the data in two. Then, each decision branch is split according to the next optimal feature until there no need for additional pruning. Once the trees are developed, each can be utilized to obtain one prediction. The final result is obtained by averaging the regression results from all decision trees. Formally, the output of the RF prediction is:

20 $${\text{y}} = \frac{1}{{n_{tree} }}\sum\limits_{i = 1}^{{n_{tree} }} {y_{i} \left( x \right)}$$

where *y* is the average output, ${\text{n}}_{tree}$ is the number of decision trees, and $y_{i} (x)$ is the individual prediction of the *i*-th tree for an input vector *x*.

#### Arithmetic optimization algorithm (AOA)

Arithmetic optimization algorithm was firstly proposed by Abualigah et al.^53^, which untilizes distribution behavior of the main arithmetic operators in mathematics. More precisely, as a new type of meta-heuristic algorithm, arithmetic functions such as subtraction(S), division (D), multiplication (M) and addition (A) have abilities to work out optimization problems without the help of derivation.

##### Solutions initialization

Normally, there are two main steps in the process of optimization: exploration and exploitation. Before that, we ought to initialize solutions. A collection of potential solution is created, as shown in Eq. (21). Each iteration leads to a better candidate solution close to the optimum solution.

21 $$X = \left[ {\begin{array}{*{20}c} {x_{1,1} } & \cdots & {x_{1,n} } \\ \vdots & \ddots & \vdots \\ {x_{N,1} } & \cdots & {x_{N,n} } \\ \end{array} } \right]$$

In order to produce the collection (*X*), the Eq. (22) can be used to identify X and shown as follows:

22 $$X = X_{LB} + rand\left( {X_{UB} - X_{LB} } \right)$$

where *rand* means randomly choosing from [0, 1], the resulting matrix is affected by the upper boundaries ($X_{UB}$) and the lower boundaries($X_{{{\text{L}}B}}$). The AOA requires a function named as Math Optimizer Accelerated (MOA) to ensure exploration and exploitation phases. The MOA is calculated in the following equation:

23 $$MOA\left( {C_{iter} } \right) = \min + C_{iter} \times \left( {\frac{\max - Min}{{M_{iter} }}} \right)$$

where $C_{iter}$ means the current iteration between 1 and the maximum number of iterations ($M_{iter}$), *MOA* ($C_{iter}$) is the function value during the iteration. Max and Min represent for the maximum and minimum values of accelerated function,respectively.

##### Exploration phase

In order to optimize process stages, consider to use MOA function. During the exploration phase, chose $r_{1}$ in random, utilize both D and M operators if *r*_1_ > MOA, during the exploration phase the AOA mainly depends on these two search strategies (D and M) and tries to find a better solution by exploring several search areas in random. On the contrary, use A and S operators if *r*_1_ < MOA. Related equation to describe the process of exploring when *r*_1_ > MOA is as follow:

24 $$x_{i,j} \left( {C_{iter} + 1} \right) = \left\{ {\begin{array}{ll} {best\left( {x_{j} } \right) \div (MOP + E) \times \left( {\left( {UB_{j} - LB_{j} } \right) \times \mu + LB_{j} } \right),} \hfill & {r_{2} < 0.5} \hfill \\ {best\left( {x_{j} } \right) \times MOP \times \left( {\left( {UB_{j} - LB_{j} } \right) \times \mu + LB_{j} } \right),} \hfill & {\hbox{otherwise}} \hfill \\ \end{array} } \right.$$

where identify a random integer between D and M operators as $r_{2}$, $\in$ is considered as a tiny value, $\mu$ is a control variable which is set to 0.5 on purpose to stable the search procedure. Besides, introduce a new parameter named as MOP (the math optimizer):

25 $$MOP\left( {C_{iter} } \right) = 1 - \frac{{{\text{C}}_{iter}^{{1/\alpha_{{\text{A}}} }} }}{{M_{iter}^{{1/\alpha_{A} }} }}$$

which $\alpha_{{\text{A}}}$ is a parameter that defines the accuracy over iterations during the exploitation.

##### Exploitation phase

Either S or A operators has produced amounts of compact data in the process of mathematical calculations, thus can apply two operators to exploitation phase. Unlike M and D operators, the A and S operators can reach the goal quicker for the reason that they have smaller diffusion. Then by trying constantly the exploitation phase will approach the best solution at last. In AOA, when the expected value of the MOA is smaller than the present value of the MOA ($C_{iter}$) under the condition of $r_{1}$, the MOA function execute S or A operator to control the exploitation investigation. We finally run out to get a better solution under the help of A and S operator by the following equation:

26 $$x_{i,j} \left( {C_{iter} + 1} \right) = \left\{ {\begin{array}{ll} {best\left( {x_{j} } \right) - MOP \times \left( {\left( {UB_{j} - LB_{j} } \right) \times \mu + LB_{j} } \right),} \hfill & {r_{3} < 0.5} \hfill \\ {best\left( {x_{j} } \right) + MOP \times \left( {\left( {UB_{j} - LB_{j} } \right) \times \mu + LB_{j} } \right),} \hfill & {\hbox{otherwise}} \hfill \\ \end{array} } \right.$$

where $r_{3}$ is an integer chosen in random to distinguish the A and S functions.

### Performance assessment

This section introduces the measures used to examine the studied methods’ performance in predicting the ground vibration. Namely, twelve statistical criteria, coefficient of determination (R^2^), mean square error (MSE), root means square error (RSME), variance account for (VAF), mean absolute error (MAE), Mean bias error(NMBE), Mean absolute percentage error (MAPE), Nash–Sutcliffe Efficiency(NS), Index of agreement (IOA), Index of scatter (IOS), a20-index (a20) and Performance index (PI) were used, and their mathematical formulae are shown in Eqs. (27)–(38)^54.^

27 $${\text{R}}^{2} = \frac{{\left[ {\sum\nolimits_{m = 1}^{n} {\left( {x_{{\text{m}}} - x_{mm} } \right)^{2} } - } \right] - \left[ {\sum\nolimits_{{{\text{m}} = 1}}^{n} {\left( {x_{m} - x_{p} } \right)^{2} } } \right]}}{{\sum\nolimits_{m = 1}^{n} {\left( {x_{m} - x_{mm} } \right)^{2} } }}$$

28 $${\text{MSE}} = \frac{1}{n}\sum\limits_{1}^{n} {\left( {x_{i} - x_{p} } \right)^{2} }$$

29 $${\text{RSME}} = \sqrt{\frac{1}{n}} \sum\limits_{i = 1}^{n} {\left( {x_{m} - x_{mm} } \right)^{2} }$$

30 $$VAF = \left( {1 - \frac{{{\text{var}} \left( {x_{m} - x_{p} } \right)}}{{{\text{var}} \left( {x_{m} } \right)}}} \right)*100$$

31 $$MAE = \frac{1}{n}\sum\limits_{i = 1}^{n} {\left( {\left| {x_{p} - x_{m} } \right|} \right)}$$

32 $$NMBE = \frac{{\frac{1}{N}\sum\nolimits_{i = - 1}^{n} {\left( {x_{p} - x_{m} } \right)^{2} } }}{{\frac{1}{N}\sum\nolimits_{i = 1}^{n} {x_{m} } }}$$

33 $$MAPE = \frac{1}{n}\sum\limits_{i = 1}^{n} {\left| {\frac{{x_{m} - x_{p} }}{{x_{m} }}} \right|*100}$$

34 $$NS = 1 - \frac{{\sum\nolimits_{i = 1}^{n} {\left( {x_{m} - x_{p} } \right)^{2} } }}{{\sum\nolimits_{i = 1}^{n} {\left( {x_{m} - x_{mm} } \right)^{2} } }}$$

35 $$IOA = 1 - \frac{{\sum\nolimits_{i = 1}^{n} {\left( {x_{p} - x_{m} } \right)} }}{{2\sum\nolimits_{i = 1}^{n} {\left( {x_{p} - x_{mm} } \right)} }}$$

36 $$IOS = \frac{RMSE}{{Avgerage\;of\;actual\;values}}$$

37 $$a20index = \frac{m20}{{N_{D} }}$$

38 $$PI = R^{2} + \left( {\frac{VAF}{{100}}} \right) - RSME$$

where ${\text{n}}$ is the number of samples, and ${\text{x}}_{m}$, ${\text{x}}_{{\text{p}}}$ and ${\text{x}}_{{{\text{mm}}}}$ denote the measured value, predicted value, and the sample mean, respectively. The m20 is the ratio of test to predicted values, and *N*_*D*_ is the total number of data samples.Theoretically, when the value of performance assessment is equal to the ideal value, , such as *R*^2^, *IOA* and NS approach 1, *MSE, RSME, MAE, NMBE, MAPE* and *IOS* approach 0, *PI* approach 2, *VAF* and a20 approach 100, it indicates the predictive model show higher performance.

## Database and feature selection

**Fig. 5:**
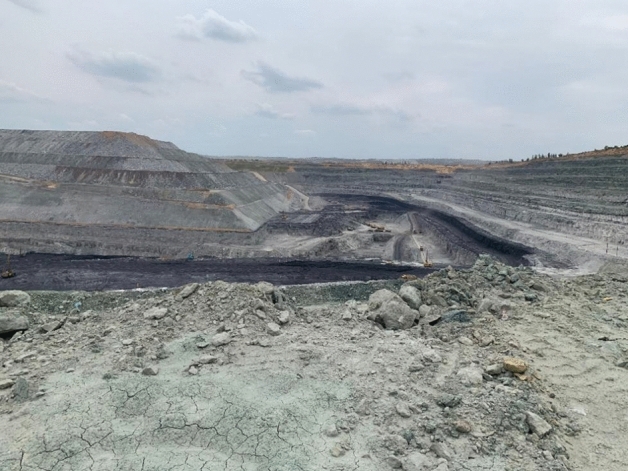
The photograph of the Wujiata coal mine.

Blasting operations is the most widely used technique for rock fragmentation and displacement in coal mines. The dataset utilized in this study was collected via the blasting tests carried at the Wujiata coal mine, located in Inner Mongolia. The area where the Wujiata coal mine is located is characterized by high topographical features in the northwest and low in the southeast (i.e., a sloped shape). Further, the topography of the area covered by aeolian sand is complex, the valleys are both vertical and horizontal, and most of them are eroded to the source. The upper part of the local geology consists of loess and a loose layer with vertical joints, due to which the joint surface collapses easily. The lower part of the rock has fewer joint cracks, and the rock quality grade is medium, so it is rated with Grade III. The studied mines are located in a close area and are similar in geological terms. Figure 5 illustrates the photograph of the Wujiata coal mine. The mine-blasting operations utilizes the 2# emulsion explosive.

**Fig. 6:**
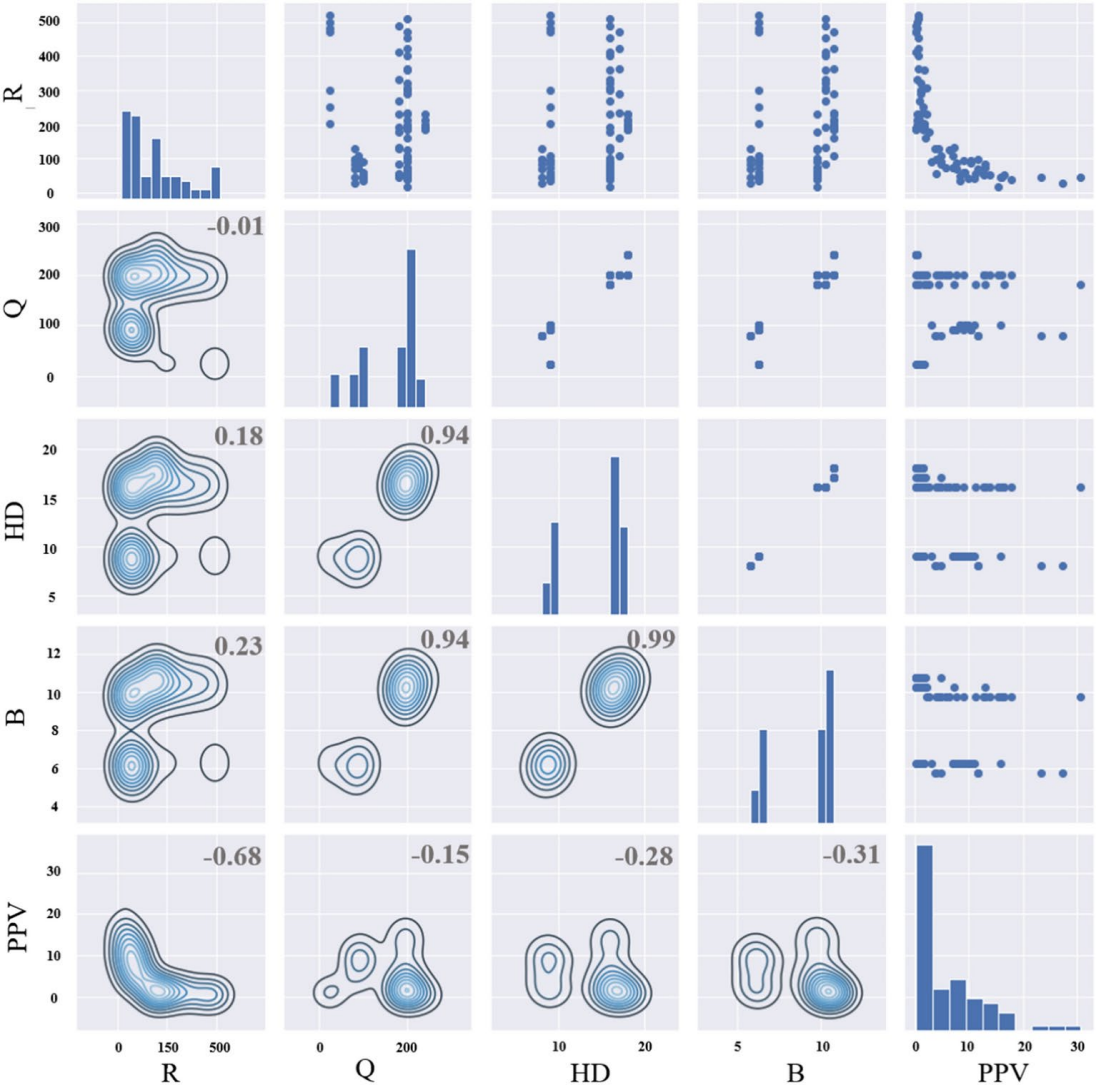
The correlations between variables.

Typically, four factor categories relevant to the ground vibration are of interest when predicting *PPV*: the distance between the blasting source and the monitored points (*R*), the blasting design, explosive parameters and geotechnical parameters. The blasting engineering parameters in this study are recorded, and the details of the dataset are listed in Table 3. Since the same site is utilized throughout this study, the hole diameter and the emulsion explosive do not vary. Thus, the influence of these parameters is not taken into account in this research. *PPV* measured in various blasting scenarios acts as an output when extracting new relationships. The relationship between two variables and the direction of correlation can be reflected in a correlation matrix plot. Pearson correlation coefficient is calculated for all variable pairs within the original data to investigate the correlation between *PPV* and the independent variables. The correlation matrix is depicted in Fig. 6, where the diagonal figure represent the variables’ distributions. The figures in the upper triangle represent the corresponding scatter points. Meanwhile, the figures below the diagonal visualize the relationship of each variable pair. Figure 6 also shows that the correlation coefficients take values of − 0.68 (between *R* and *PPV*), − 0.15 (*Q* and *PPV*), − 0.28 (*HD* and *PPV*), and − 0.31 (*B* and *PPV*) at the left-upper corner of the upper triangle. The correlation coefficient between *R* and *PPV* is the closest to − 1, indicating that the influence of *R* on predicting *PPV* is more pronounced than that of the remaining parameters.

**Table 3:** The details of recorded dataset.

	R	Q	HD	B	PPV
Mean	184.82	156.85	13.94	8.85	5.97
Standard error	16.01	7.18	0.43	0.22	0.73
Median	130.47	190.00	16.00	9.75	3.50
Standard deviation	143.22	64.25	3.80	2.00	6.56
Sample variance	20,511.90	4127.57	14.46	3.98	42.99
Kurtosis	− 0.29	− 0.72	− 1.46	− 1.49	2.41
Skewness	0.92	− 0.78	− 0.59	− 0.60	1.53
Minimum	17.93	24.00	8.00	5.75	0.34
Maximum	518.45	240.00	18.00	10.75	30.69
CL (95%)	31.38	14.08	0.83	0.44	1.44

## Results

### Prediction based on empirical equations

As noted in previous sections, many researchers utilized empirical equations for predicting PPV. This section compares the most widely used equations (mentioned in Table 1) regarding the accuracy of their predictions. Furthermore, the accuracy of the new empirical equation that considers burden (i.e., Eq. 7) is assessed. Based on the parameters utilized in each empirical equation, the correlation data are selected for regression analysis. The constants employed in empirical equations were determined via linear or non-linear regression analysis. Similar to the ML models, 80% of the dataset was used to train empirical equations, and the remaining 20% served as the testing set.

**Fig. 7:**
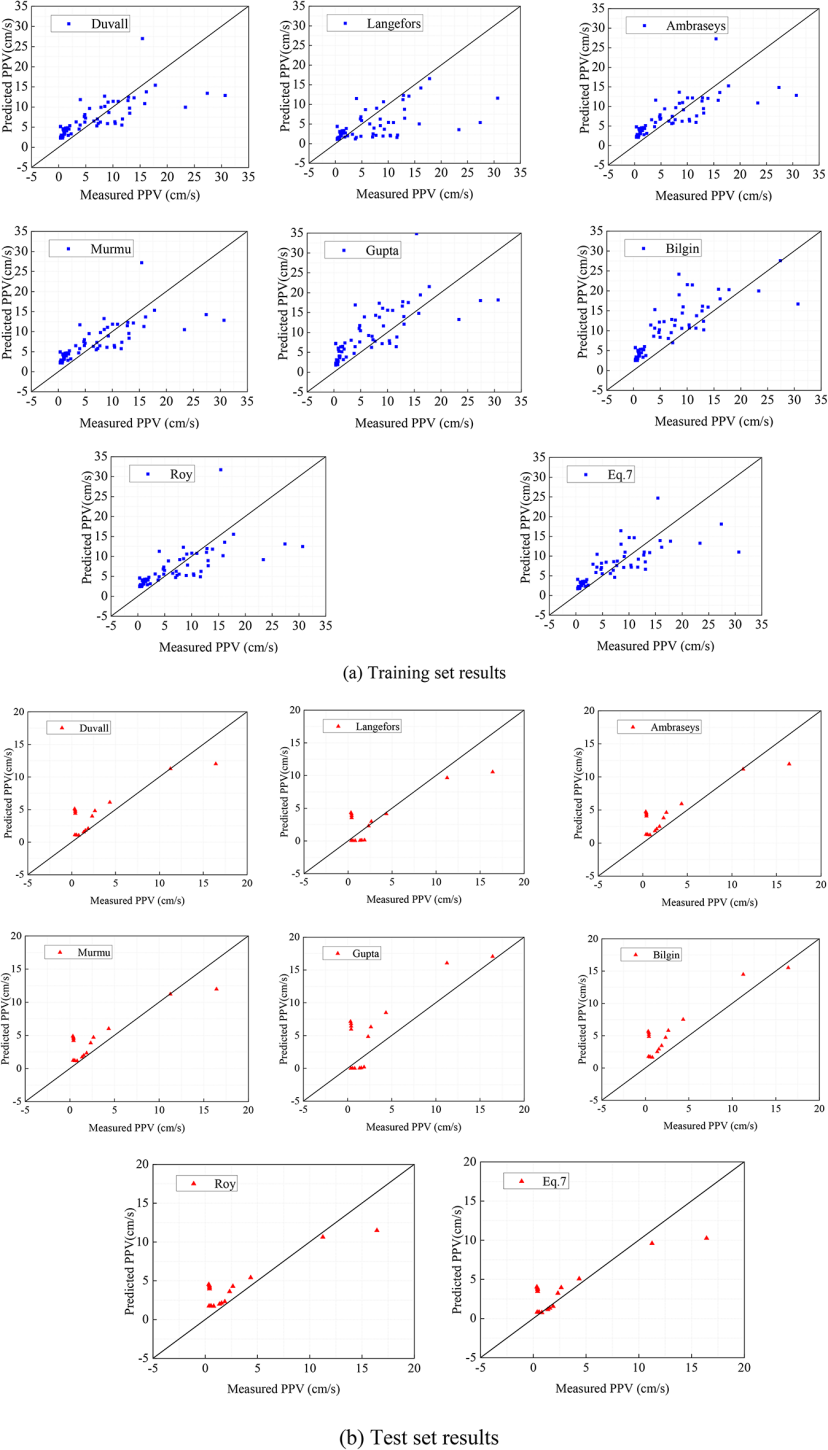
Comparison of measured PPV and predicted PPV using empirical equations.

Since this section aims to compare the empirical equations for predicting PPV regarding the best fit, Table 4 lists the constants calculated via regression analysis. Figure 7 shows the relationship between measured and predicted PPV for all empirical equations. Table 5 further lists the performance assessments regarding the predictions to enable evaluating the empirical models’ accuracy. Based on Table 5, Eq. (7) shows the better performance than other empirical equations, and yields the R^2^, MSE, RSME, MAE, NMBE, MAPE, NS, IOA IOS and PI values of 0.6, 18.12, 4.26, 3.03, 2.68, 130.61, 0.6, 0.85, 0.63 and − 3.65 on the train set, respectively. Meanwhile, Eq. (7) yields the values of R^2^, MSE, RSME, VAF, MAE,NMBE, MAPE, NS, IOS, a20 and PI of 0.71, 5.64, 2.38, 0.72, 1.63, 1.98 244.01, 0.71, 0.83, 37.50 and − 1.66 on the test set, repsectively.

**Table 4:** The constants computed via regression analysis.

Empirical model	Constants		
	*k*	$\alpha$	Other
Duvall	32.11	0.74	–
Langefors	0.21	3.05	–
Ambraseys	64.20	0.76	–
Murmu	48.45	0.75	$\beta = 0.005$
Gupta	41.41	0.58	–
Bilgin	319	0.79	$\beta = 0.39$
Roy	1.36	38.49	$\gamma = - 0.86$
Equation (7)	116.8	2.13	$\beta = - 1.36$

**Table 5:** The performance assessment of different empirical equations.

Empirical model	Phase	R^2^	MSE	RSME	VAF	MAE	NMBE	MAPE	NS	IOA	IOS	a20	PI
Duvall	Train	0.53	21.33	4.62	0.53	3.33	3.16	175.73	0.53	0.80	0.68	20.31	− 4.08
	Test	0.65	6.79	2.60	0.74	1.89	2.38	325.27	0.65	0.89	0.91	25.00	− 1.95
Langefors	Train	0.10	40.61	6.37	0.17	4.02	6.02	117.81	0.10	0.68	0.94	15.63	− 6.27
	Test	0.69	6.04	2.46	0.69	1.81	2.12	274.38	0.69	0.89	0.86	25.00	− 1.77
Ambraseys	Train	0.56	19.84	4.45	0.57	3.22	2.94	163.31	0.56	0.82	0.66	20.31	− 3.89
	Test	0.68	6.06	2.46	0.77	1.88	2.12	314.32	0.68	0.90	0.86	6.25	− 1.77
Murmu	Train	0.55	20.42	4.52	0.55	3.26	3.02	167.93	0.55	0.82	0.67	21.88	− 3.96
	Test	0.67	6.31	2.51	0.76	1.88	2.21	317.61	0.67	0.90	0.88	18.75	− 1.83
Gupta	Train	0.35	29.31	5.41	0.51	4.27	4.34	220.65	0.35	0.81	0.80	12.50	− 5.06
	Test	0.28	13.89	3.73	0.51	2.95	4.87	465.33	0.28	0.86	1.31	6.25	− 3.44
Bilgin	Train	0.25	34.02	5.83	0.55	4.38	5.04	215.10	0.25	0.82	0.86	21.88	− 5.58
	Test	0.53	9.03	3.00	0.84	2.57	3.16	409.16	0.53	0.89	1.05	6.25	− 2.47
Roy	Train	0.47	23.84	4.88	0.47	3.36	3.53	164.28	0.47	0.79	0.72	25.00	− 4.41
	Test	0.69	6.05	2.46	0.77	1.96	2.12	324.71	0.69	0.89	0.86	18.75	− 1.77
Equation (7)	Train	0.60	18.12	4.26	0.60	3.03	2.68	130.61	0.60	0.85	0.63	17.19	− 3.65
	Test	0.71	5.64	2.38	0.72	1.63	1.98	244.01	0.71	0.89	0.83	37.50	− 1.66

Based on the performance assessment, the score anlysis is adopted to find the effectiveness of empirical equations. Eight empirical equations are taken into account in this analysis, the score of “J” is 8 which has obtained the best value for each equation. The higher score, calculated independently for training and testing results, indicates the best value for the same performance assessment. Finally, the obtained scores across either training or testing databases are added to determine the final score. Table 6 lists the scores of twelve performance assassments in the training and test database. Figure 6 illustrates that the Eq. (7) shows the best performance in both training and testing database, which indicates Eq. (7) presents the greatest performance in predicting PPV. In particular, Eq. (7) obtains higher score when predicting PPV, other empirical equations generate less accurate predictions. In general, the prediction accuracy of empirical equations is not high.

**Table 6:** The score analysis of empirical equations.

Empirical model	R^2^	MSE	RSME	VAF	MAE	NMBE	MAPE	NS	IOA	IOS	a20	PI	Total
Train													
Duvall	5	5	5	4	5	5	3	5	3	5	5	5	55
Langefors	1	1	1	1	3	1	8	1	1	1	2	1	22
Ambraseys	7	7	7	7	7	7	6	7	7	7	4	7	80
Murmu	6	6	6	6	6	6	4	6	5	6	7	6	70
Gupta	3	3	3	3	2	3	1	3	4	3	1	3	32
Bilgin	2	2	2	5	1	2	2	2	6	2	6	2	34
Roy	4	4	4	2	4	4	5	4	2	4	8	4	49
Equation (8)	8	8	8	8	8	8	7	8	8	8	3	8	90
Test													
Duvall	3	3	3	4	4	3	3	3	3	3	7	3	42
Langefors	7	7	7	2	7	7	7	7	6	7	6	7	77
Ambraseys	5	5	5	7	5	5	6	5	8	5	3	5	64
Murmu	4	4	4	5	6	4	5	4	7	4	5	4	56
Gupta	1	1	1	1	1	1	1	1	1	1	2	1	13
Bilgin	2	2	2	8	2	2	2	2	2	2	1	2	29
Roy	6	6	6	6	3	6	4	6	5	6	4	6	64
Equation (8)	8	8	8	3	8	8	8	8	8	8	8	8	91

### ML models’ performance

This section compares the performances of six ML models obtained using different algorithms. To analyze each algorithm’s feasibility, the workflow shown in Fig. 2 is followed, and the accuracy of PPV predictions is evaluated. Specifically, the performance assessment of each ML methods on both training and testing sets are calculated. The performance assessments of each ML model on both the training and tests set are performed in Table 7. Table 7 illustrates that RF and KNN show better performance in predicting PPV than other ML models. RF yields the R^2^ , MSE, RSME, VAF, MAE, NMBE, MAPE, NS, IOA IOS and PI values of 0.95, 2.41, 1.55, 0.95, 0.92, 0.36, 18.16, 0.95, 0.99, 0.23,68.75 and -0.60 on the train set, respectively. Meanwhile, RF yields the R^2^ , MSE, RSME, VAF, MAE, NMBE, MAPE, NS, IOA IOS and PI values of 0.98, 0.38, 0.61, 0.98, 0.46, 0.13, 47.42, 0.98, 0.99, 0.21, 31.25 and 0.38, respectively.

**Table 7:** The performance assessment of machine learning models.

Empirical model	Phase	R^2^	MSE	RSME	VAF	MAE	NMBE	MAPE	NS	IOA	IOS	a20	PI
ANN	Train	− 0.93	87.29	9.34	− 0.70	7.65	12.93	693.22	− 0.93	0.41	1.38	10.94	− 10.28
	Test	− 28.54	567.99	23.83	− 13.18	17.33	198.99	2824.29	− 28.54	0.11	8.35	6.25	− 52.51
KNN	Train	0.78	9.77	3.12	0.79	1.98	1.45	40.95	0.78	0.93	0.46	31.25	− 2.33
	Test	0.82	3.55	1.88	0.84	1.18	1.24	77.43	0.82	0.96	0.66	31.25	− 1.06
LR	Train	0.47	24.06	4.91	0.47	3.63	3.56	199.13	0.47	0.79	0.73	20.31	− 4.43
	Test	0.29	13.59	3.69	0.39	3.42	4.76	428.45	0.29	0.79	1.29	6.25	− 3.39
RF	Train	0.95	2.41	1.55	0.95	0.92	0.36	18.16	0.95	0.99	0.23	68.75	− 0.60
	Test	0.98	0.38	0.61	0.98	0.46	0.13	47.42	0.98	0.99	0.21	31.25	0.38
RR	Train	0.48	23.73	4.87	0.48	3.56	3.52	194.62	0.48	0.79	0.72	20.31	− 4.39
	Test	0.16	16.05	4.01	0.16	3.64	5.62	539.86	0.16	0.78	1.40	6.25	− 3.84
SVR	Train	0.48	23.63	4.86	0.52	2.77	3.50	63.18	0.48	0.75	0.72	28.13	− 4.38
	Test	0.66	6.46	2.54	0.68	1.98	2.26	215.37	0.66	0.85	0.89	12.50	− 1.87

**Fig. 8:**
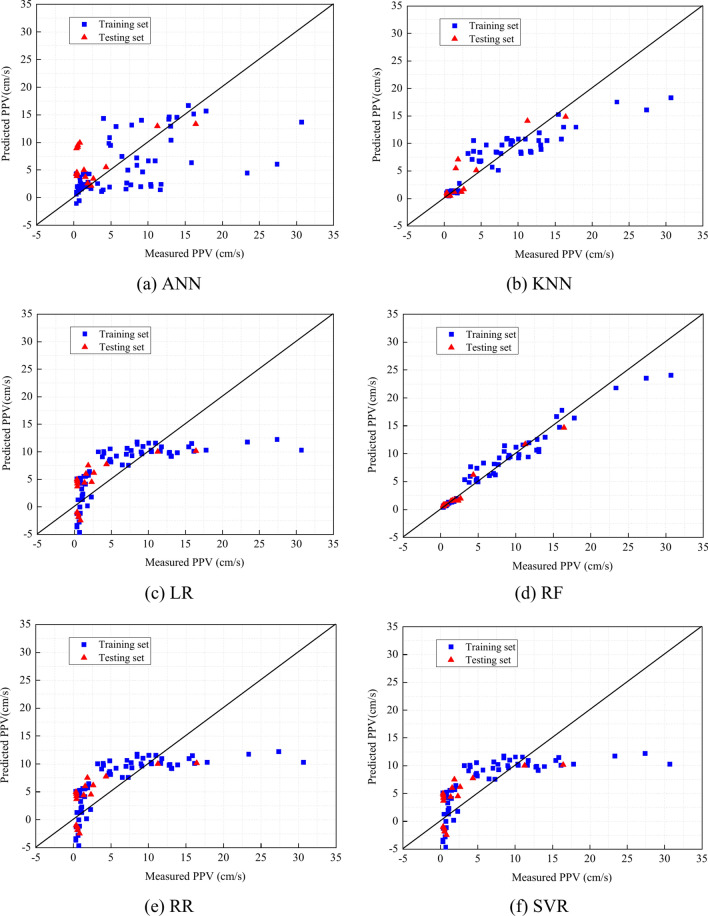
Comparison of measured PPV and PPV predicted using ML algorithms.

**Fig. 9:**
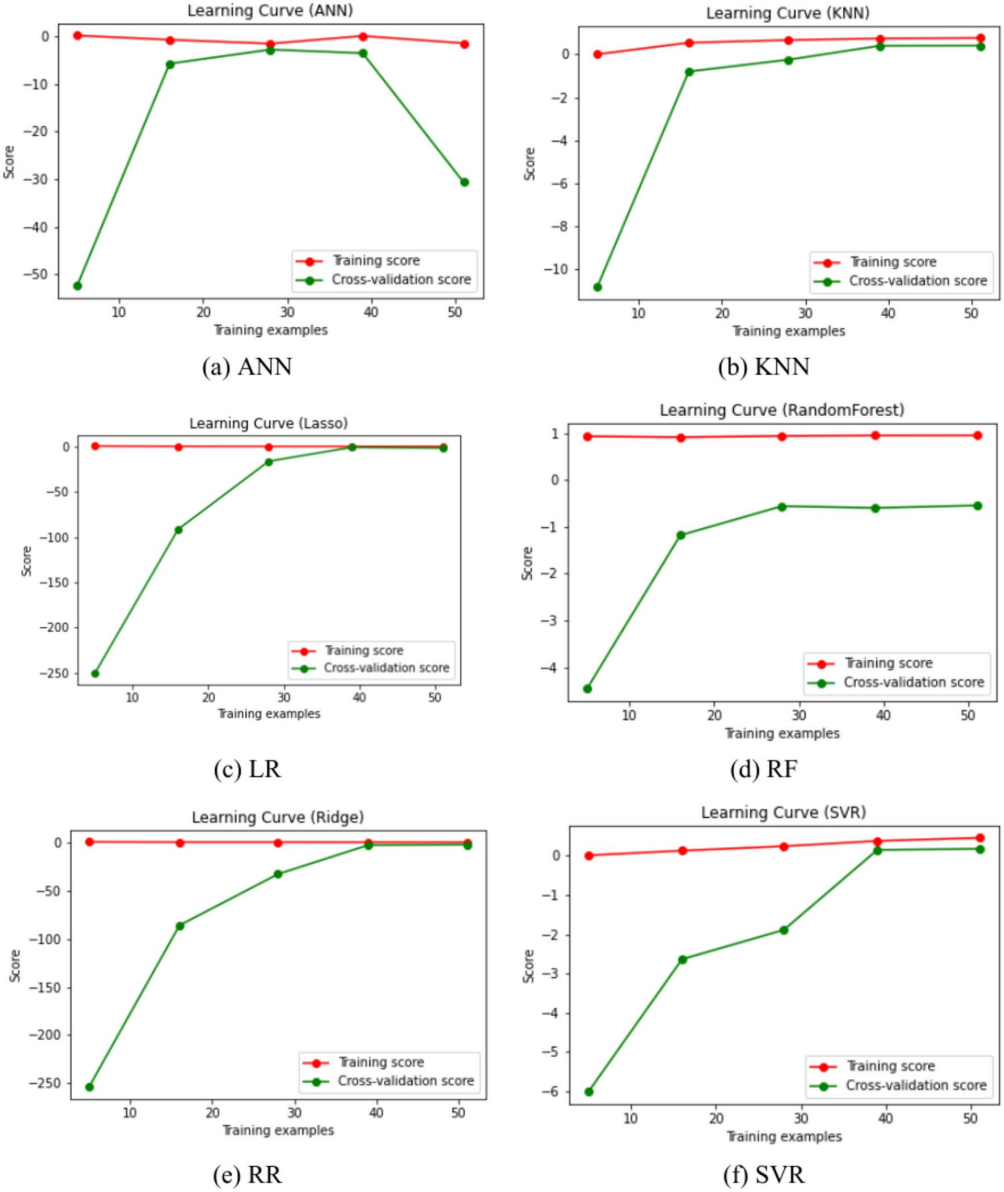
Learning curves of ML algorithms.

As shown in Fig. 8, the RF’s and KNN’s predictions are close to the identity line, indicating that their performances are better than those of the other four models. According to Fig. 9, the ANN, Lasso, and Ridge model might be overfitting since the cross-validation score decreases after a certain point. SVR seems to be learning well but might require more data or parameter tuning for better performance. Similar to SVR, the KNN model shows high variance with limited data, but it improves significantly with more data. The RF model appears to have the best generalization so far, with high and stable training scores and improving cross-validation scores.

**Fig. 10:**
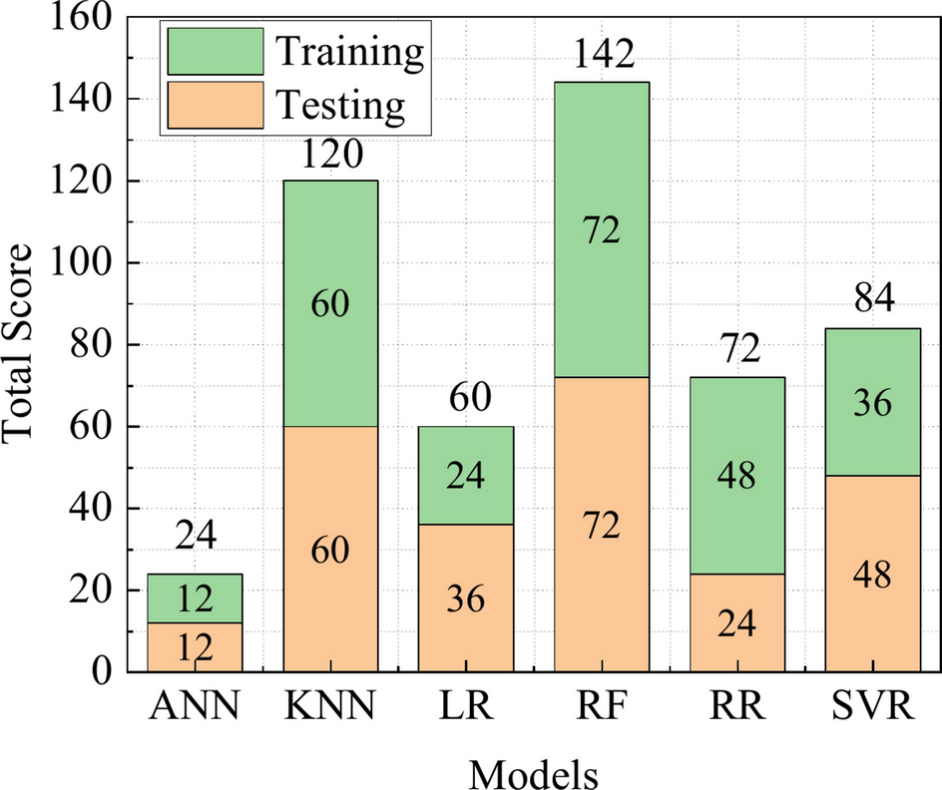
The score analysis of ML models.

To further verify the prediction accuracy, the score analysis is also adopted and listed in Table 8. To exhibit the scores of ML models, as shown in Fig. 10, the models’ predictions are judged by whether their scores are larger than other ML models, respectively. It can be seen from Fig. 10 that out of all models, RF’s score is the largest than the scores of other ML models in both training and testing datasets. On both training and testing sets, KNN demonstrates a nearly perfect performance, and performs better than RR, LR, ANN, and SVR. These results show that RF and KNN are suitable for predicting PPV. According to the workflow, these two best-performing algorithms are selected for further optimization, while RR, LR, ANN, and SVR failed to enter the next analysis round.

**Table 8:** The score analysis of machine learning models.

Empirical model	R^2^	MSE	RSME	VAF	MAE	NMBE	MAPE	NS	IOA	IOS	a20	PI	Total
Train													
ANN	1	1	1	1	1	1	1	1	1	1	1	1	12
KNN	5	5	5	5	5	5	5	5	5	5	5	5	60
LR	2	2	2	2	2	2	2	2	2	2	2	2	24
RF	6	6	6	6	6	6	6	6	6	6	6	6	72
RR	4	4	4	4	4	4	4	4	4	4	4	4	48
SVR	3	3	3	3	3	3	3	3	3	3	3	3	36
Test													
ANN	1	1	1	1	1	1	1	1	1	1	1	1	12
KNN	5	5	5	5	5	5	5	5	5	5	5	5	60
LR	3	3	3	3	3	3	3	3	3	3	3	3	36
RF	6	6	6	6	6	6	6	6	6	6	6	6	72
RR	2	2	2	2	2	2	2	2	2	2	2	2	24
SVR	4	4	4	4	4	4	4	4	4	4	4	4	48

#### Regression error characteristics (REC) curve

**Fig. 11:**
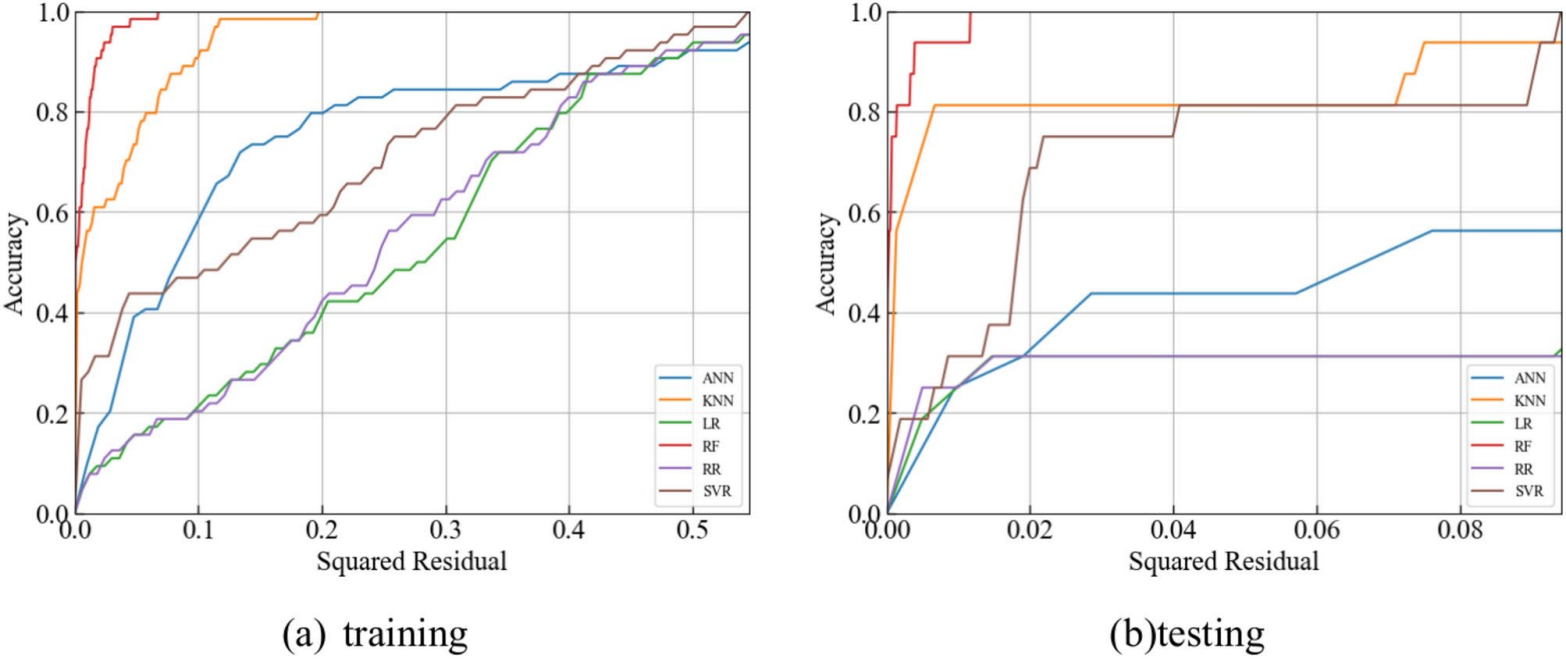
REC curve for machine learning models.

The Regression Error Characteristic (REC) curve is a diagnostic tool in regression analysis that evaluates predictive model accuracy^55,56^. This graphical representation of cumulative prediction error distribution aids in assessing model performance across various error thresholds. The REC curve plots error tolerance on the x-axis against the percentage of predictions within that tolerance on the y-axis. Each point on the curve corresponds to a specific error tolerance, indicating the proportion of predictions with errors within that threshold. To compare the performance of each model, the REC curves for different models have been plotted, as shown in Fig. 11. Figure 11 illustrates the REC curves for ML models. Meanwhile, Table 9 lists the AOC values which represents the area over the REC curves. The smaller value of AOC, the better performance of ML models in predicting PPV. Therefore, RF model has less AOC value than other ML models in predicting PPV.

**Table 9:** The AOC values of different ML models.

	Actual	ANN	KNN	LR	RF	RR	SVR
Training	0	0.79	0.17	0.34	0.06	0.35	0.38
Tesing	0	0.65	0.11	0.28	0.01	0.26	0.07

#### Uncertainty analysis (UA)

UA is a method used to quantify and assess the uncertainty in model prediction results, especially applicable to scenarios requiring accurate predictions, such as predicting PPV. By analyzing how uncertainties in input parameters propagate to the outputs, UA determines the prediction errors of the model and provides a more comprehensive evaluation of the model’s reliability^57^. UA compares the predicted results with actual data to assess the model’s reliability and accuracy. UA^54^ involves calculating multiple statistical indicators such as absolute error, Margin of Error (MOE), standard deviation (SD_ev_), standard error (SE), Margin of Error at a 95% confidence level (ME), white blood cell count (WBC), upper bound (UB), and lower bound (LB). The calculation of these indicators not only reveals the distribution of prediction errors during the training and testing phases but also helps clarify the model’s reliability in practical applications. In documenting research results, models with low WBC values are typically considered to have higher prediction accuracy and reliability. The UA results for 6 ML models are listed in Table 10. Based on the WBC of models, RF and KNN are suggested to predict the PPV in the nest round.

**Table 10:** Results obtained from the UA.

Models	MOE	SD_ev_	SE	ME	LB	UB	WBC	Rank
Training								
ANN	21.73	8.84	1.10	2.17	12.14	7.80	4.33	6
KNN	12.40	3.13	0.39	0.77	7.17	5.63	1.53	2
LR	20.40	4.94	0.62	1.21	7.96	5.54	2.42	5
RF	6.61	1.56	0.20	0.38	7.09	6.32	0.77	1
RR	20.89	4.91	0.61	1.20	7.95	5.55	2.41	4
SVR	21.85	4.68	0.59	1.15	6.46	4.17	2.29	3
Testing								
ANN	46.06	17.05	4.26	8.36	28.40	11.68	16.71	6
KNN	5.21	1.83	0.46	0.90	4.39	2.60	1.79	2
LR	6.28	3.54	0.88	1.73	5.95	2.48	3.47	4
RF	1.65	0.61	0.15	0.30	3.33	2.74	0.59	1
RR	6.79	4.14	1.03	2.03	4.91	0.85	4.06	5
SVR	7.75	2.56	0.64	1.25	4.69	2.18	2.50	3

### The improved models’ performance

**Fig. 12:**
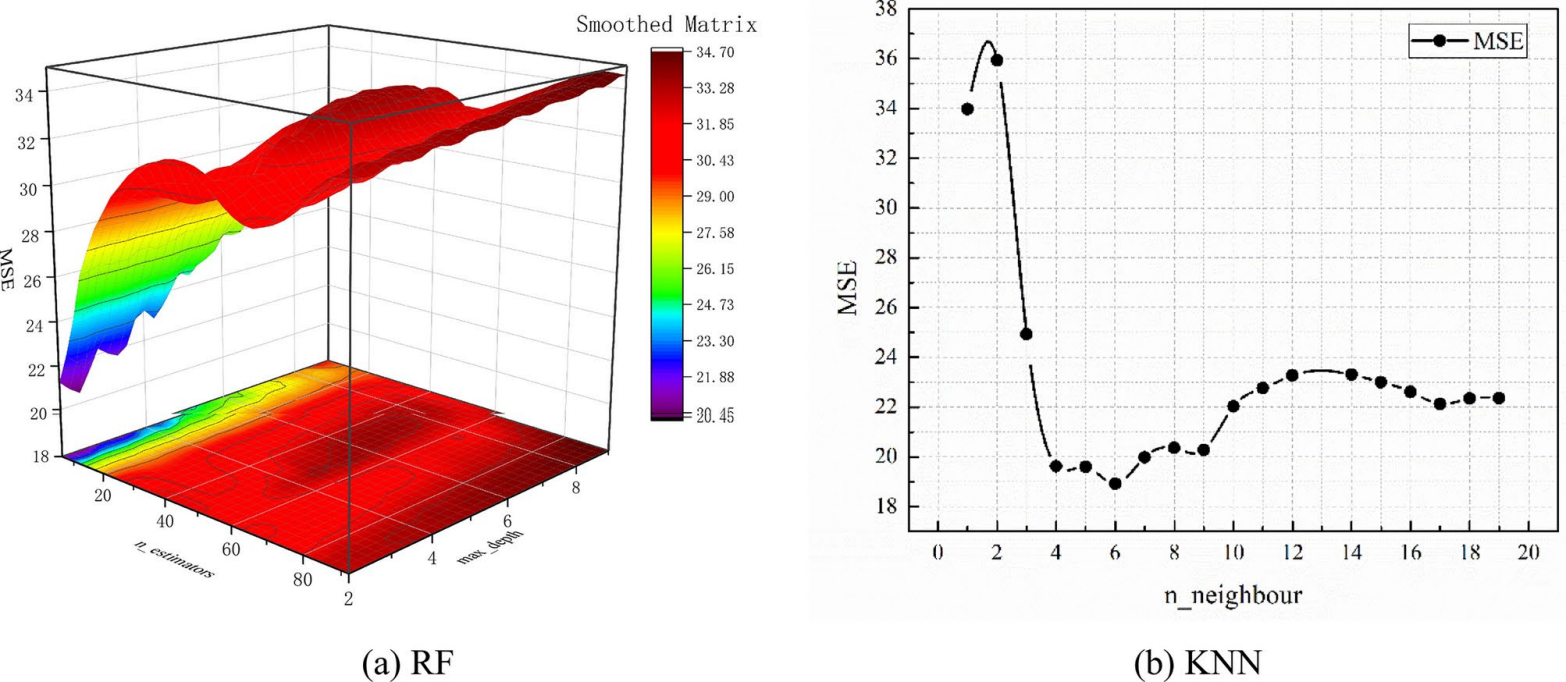
Results of the AOA algorithm for hyper-parameters.

According to results presented in section “ML models’ performance”, RF and KNN are better at predicting PPV than the other considered models. Next, the AOA algorithm is utilized to optimize the models’ performance and achieve higher accuracy. The AOA algorithm aims to select the optimal values of hyper-parameters in RF and KNN algorithms via a systematic analysis of their performances for various parameter values. The considered parameter values are derived from a certain range using a specified step distance. In this research, the values of ‘n_estimator’ (i.e., number of trees) and ‘max depth’ (i.e., the maximum tree depth) in RF and the value of ‘n_neighbour’ in KNN are searched for training the two models. Figure 12 shows the test performance of the two models when considering different hyper-parameter values. Figure 12a reveals a high-MSE region for RF with ‘n_estimator’ being 8 and ‘max depth’ being 5. Meanwhile, the high-MSE region for KNN is obtained when ‘n_neighbour’ is 5 (in Fig. 12b). The models with hyper-parameter sets in the identified regions provide high-accuracy predictions of actual PPV.

**Fig. 13:**
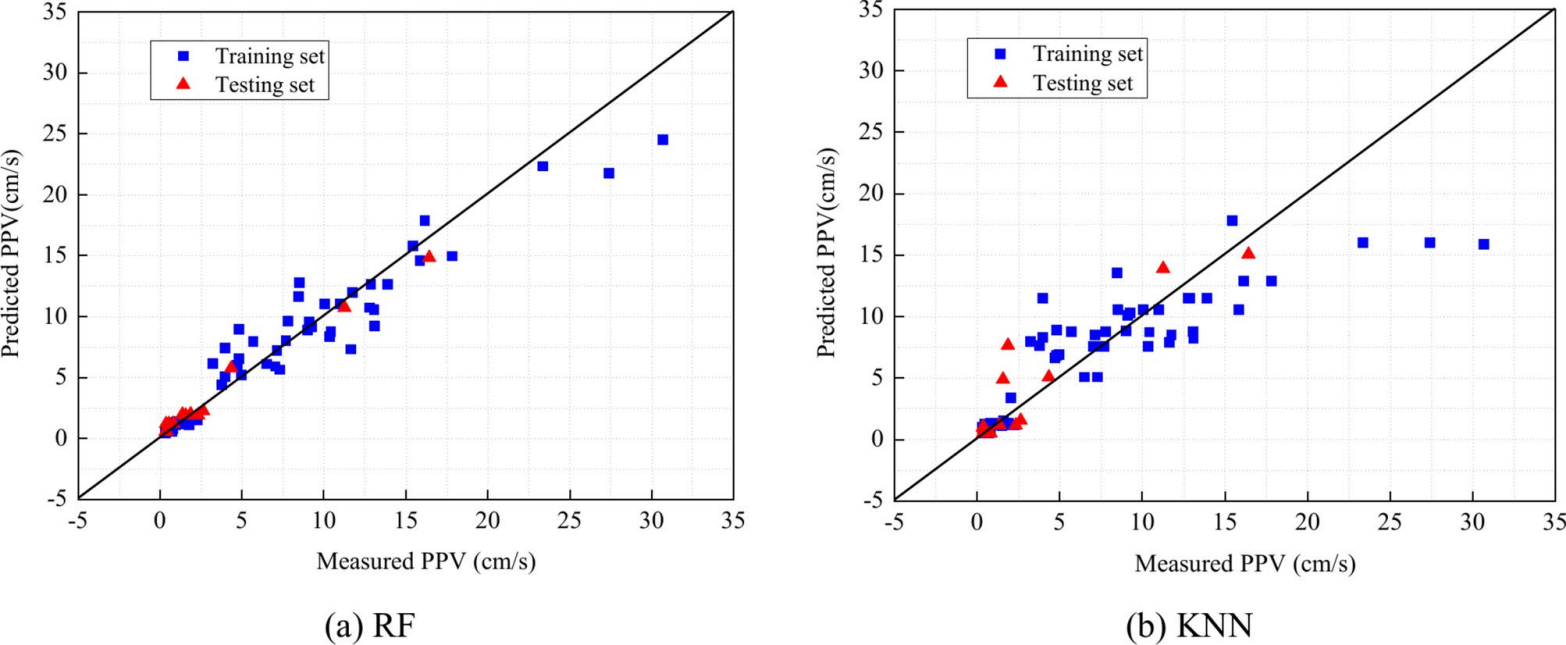
Comparison between measured and predicted PPV with optimum hyper-parameters.

Finally, the two optimal algorithms and their performance assessment are shown in Fig. 13. Simultaneously, the figure presents the comparison between measured and predicted PPVs. To compare the predictive performance of opitimzed RF and KNN, performance assessments of the models are summarized in Table 11. For the training and testing sets, AOA-RF shows perfect agreement with the measured PPV, yielding the better performance than AOA-KNN. In accordance, the majority of the points are close to the identity line.

**Table 11:** The performance assessment of AOA-RF and AOA-KNN.

Models	Training		Testing	
	AOA-RF	AOA-KNN	AOA-RF	AOA-KNN
R^2^	0.92	0.74	0.98	0.81
MSE	3.52	11.81	0.47	3.59
RSME	1.88	3.44	0.68	1.89
VAF	92.25	74.26	97.72	83.52
MAE	1.19	2.11	0.54	1.18
NMBE	0.52	1.75	0.16	1.26
MAPE	24.91	41.69	53.04	80.37
NS	0.92	0.74	0.98	0.81
IOA	0.98	0.91	0.99	0.96
IOS	0.28	0.51	0.24	0.66
a20	51.56	37.50	31.25	37.50
PI	− 0.94	− 2.69	0.30	− 1.07

**Fig. 14:**
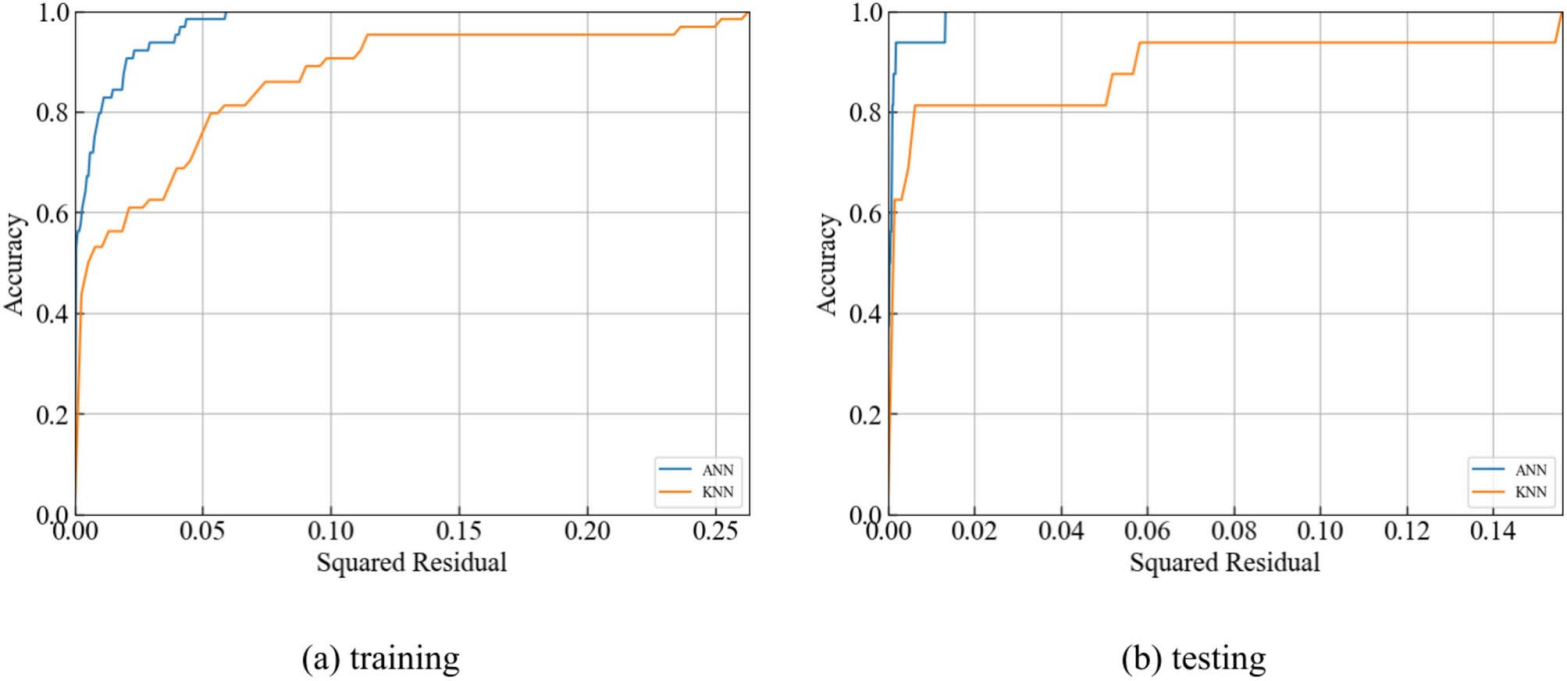
REC curve for opitimized ML models.

Meanwhile, the REC curves for two opitimzed models are plotted in Fig. 14. The training AOCs calculated for AOA-RF and AOA-KNN are 0.05 and 0.23, respectively. And the testing AOCs calculated for AOA-RF and AOA-KNN are 0.01 and 0.14, respectively. The results reveals that AOA-RF predicts PPV to be better AOC, close to the AOC of measured PPV.

UA is also calculated for evaluating the performance of opitimzed models, and the results of AOA-RF and AOA-KNN are recorded in Table 11. According to the Table 12, AOA-RF both for training and testing datasets gets the higher position than AOA-KNN, which indicates that AOA-RF. Based on these results, RF also achieves a good performance on a training set. However, compared with RF, the KNN performance is worse. Thus, combined with the above results, these results indicate the RF’s suitability for predicting PPV.

**Table 12:** Results obtained from the UA.

Models	MOE	SD_ev_	SE	ME	LB	UB	WBC	Rank
Training								
AOA-RF	6.20	1.89	0.24	0.46	7.10	6.18	0.92	1
AOA-KNN	14.78	3.44	0.43	0.84	7.18	5.50	1.69	2
Testing								
AOA-RF	1.59	0.68	0.17	0.33	3.36	2.69	0.67	1
AOA-KNN	5.77	1.84	0.46	0.90	4.40	2.60	1.80	2

**Fig. 15:**
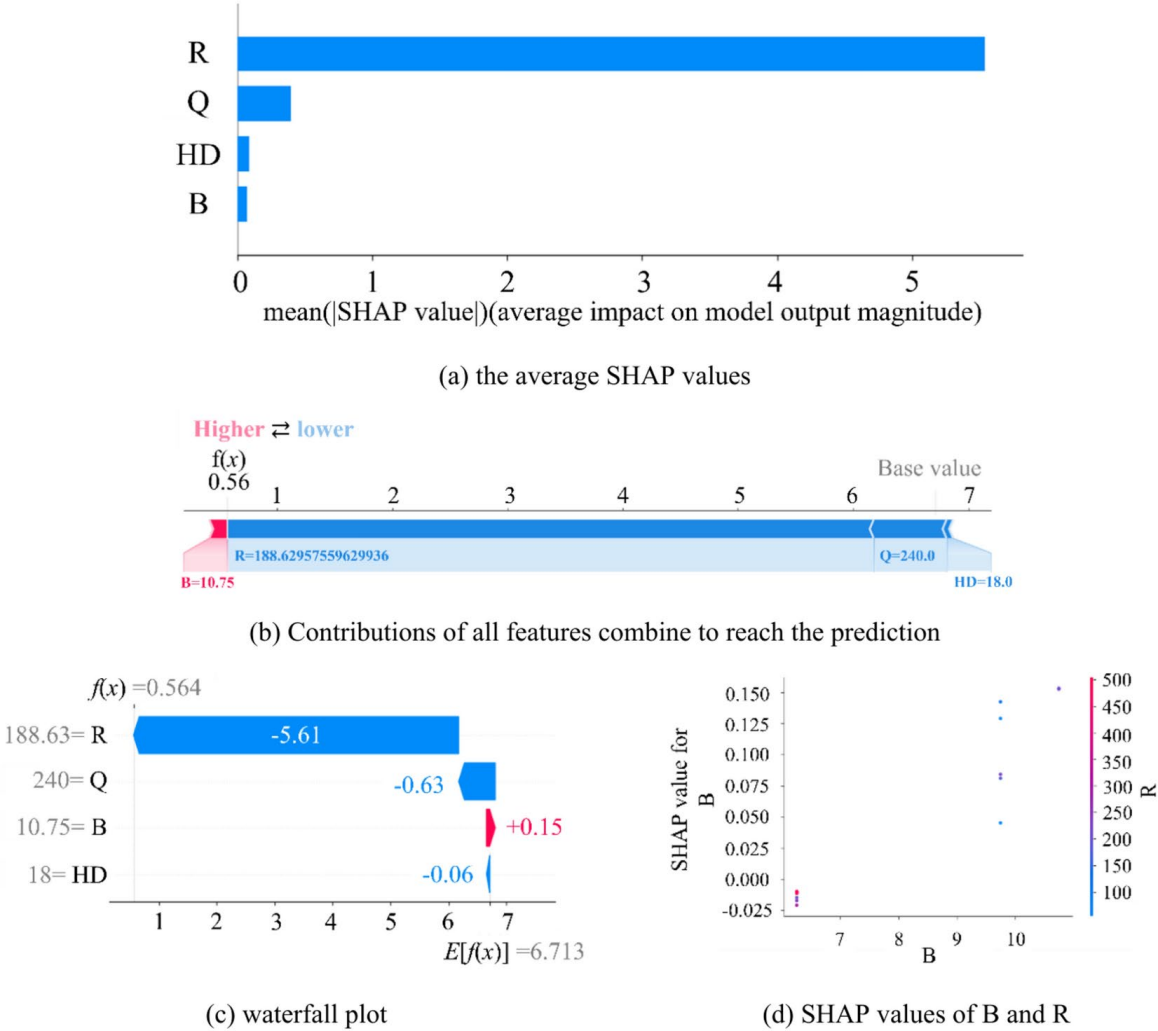
ML-based feature importance from the AOA-RF model based on SHAP values.

To investigate the parameters on the feature contributions in the AOA-RF model, SHapley Additive explanations (SHAP) is used to help build a solid understanding of how to compute and interpret SHAP of the AOA-RF model trained with the dataset and quantitatively evaluate feature importance for predicting PPV. As shown in Fig. 15a, the SHAP summary plot shows that the average SHAP values (absolute value) for all features across the test set, ‘R’ generally holds the most importance in the model as it possesses the highest average SHAP value, while the average importance of the other features is lower. From Fig. 15b,c, figures indicate that burden is positively correlated with the predicted results, and other three parameters are nagetively corelated with PPV. In Fig. 15d, a SHAP dependence plot displays the relationship between the SHAP values of B and R, and shows how the impact of ‘B’ on the model’s prediction changes as the values of ‘R’ vary. In summary, R is a key factor that significantly contributes to the prediction. Burden, while still impactful, have a lesser influence in comparison.

## Discussion and outlook

### Disucssion

This research summarized and compared many empirical equations and ML algorithms to identify the response mechanism for predicting the ground vibration. Similar to several earlier studies, this work considers multiple parameters in an empirical equation, but the obtained empirical accuracy is worse than that achieved via ML algorithms. The main reason lies in the difficulty of considering all influential parameters in an empirical equation. Further, one can note that linear regression algorithms such as RR and LR are not suitable for solving non-linear problems. Meanwhile, the SVR’s performance was suboptimal within this work. On the other hand, RF algorithm accurately capture the evolution of actual observations with lower errors, indicating the integrated algorithm’s suitability for PPV prediction.

**Fig. 16:**
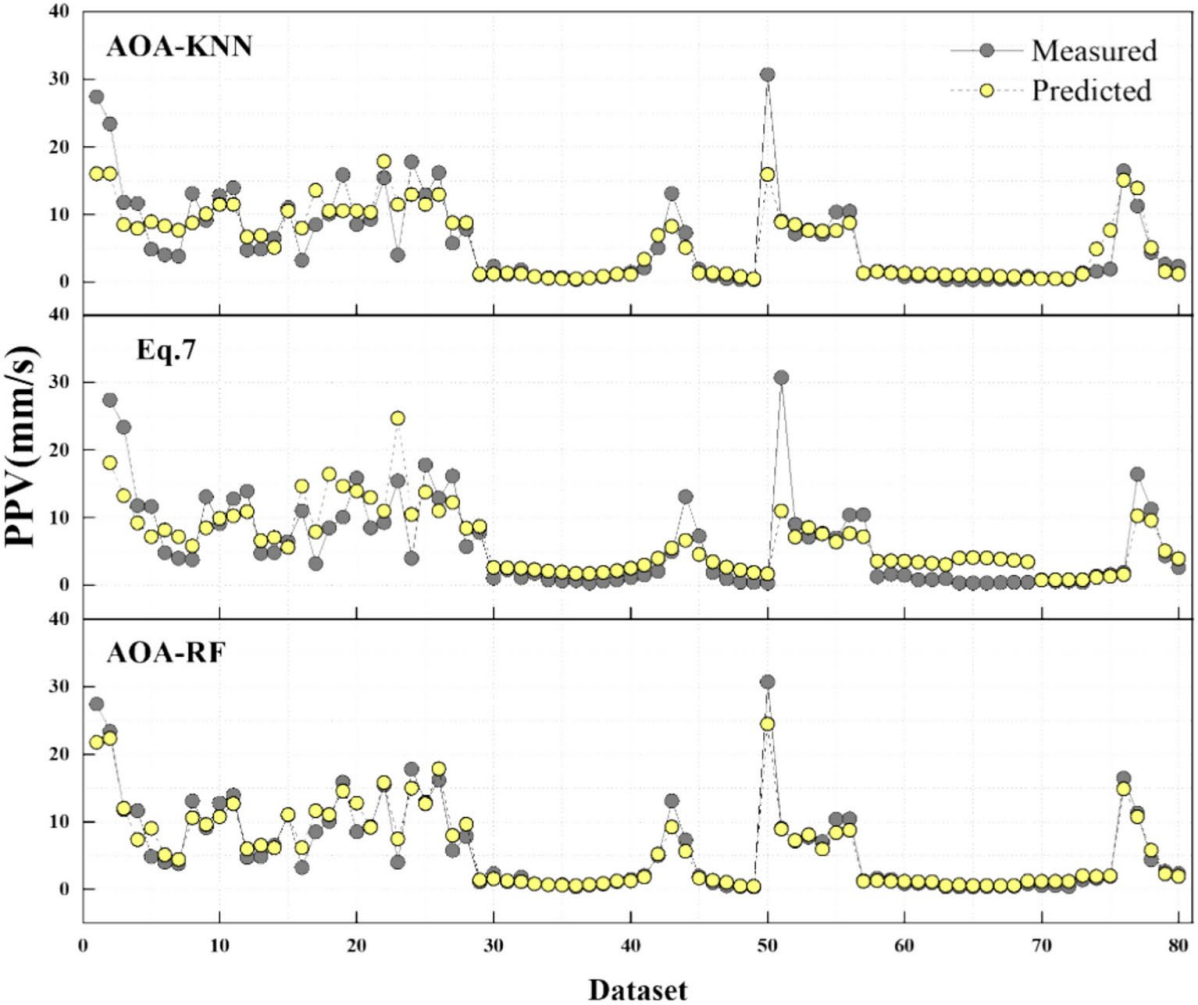
The four models’ PPV prediction results.

To analyze the performance gap between empirical equations and ML algorithms, the evolutions of the predicted PPV using two best-performing empirical equations and two best-performing ML algorithms are shown in Fig. 16 and compared to the actual PPV. Figure 16 demonstrates that RF achieves the best performance in predicting PPV. RF yields the values of performance assessments, AOC and UA superior to those obtained using empirical equations. Thus, one can note that, overall, ML algorithms outperform empirical equations in predicting PPV.

**Fig. 17:**
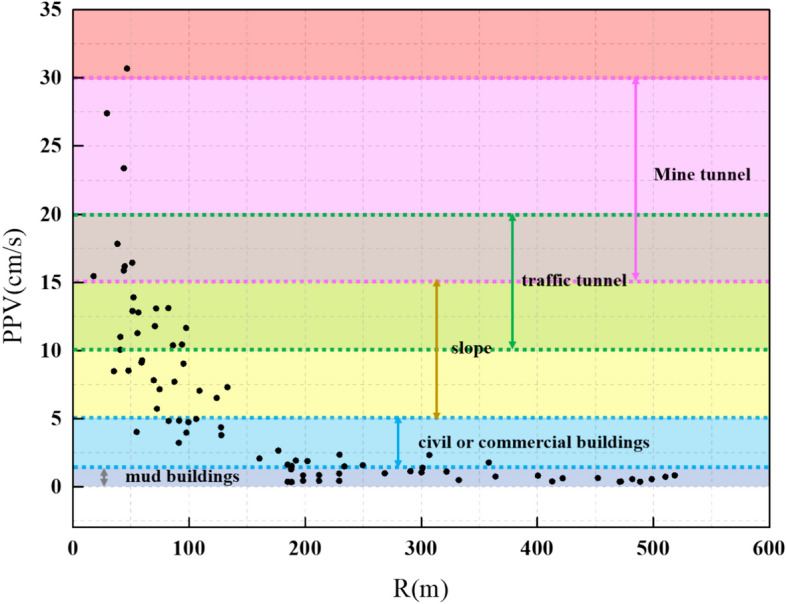
Disaster warning for different building types.

To prevent the damage of surrounding urban communities, it is necessary to determine the dangerous velocity of blasting vibration for various common buildings. The PPV values greater than the limit for different building types are extracted based on “Safety Regulations for Blasting (GB6722-2014)” and marked in Fig. 17. Thus, Fig. 17 provides a disaster warning for the surrounding urban communities and lets the engineers optimize the blasting design to reduce the PPV.

### Limitation and outlook

The presented research demonstrates that ML can be utilized as an approach to predicting PPV. However, compared with empirical equations, ML algorithms typically require significantly more datasets to obtain accurate predictions. The competive ML models only relied on data from the Wujiata coal mine. This location-specific data means the results may not generalize well to other sites with different geological conditions or blasting designs. Although the RF model shows good performance in predicting PPV, certain influential parameters, such as geological mechanics parameters, the detonation velocity of explosives and temperature are not considered.These results can only be applied to geological conditions similar to this engineering study.

Therefore, further studies should expand the dataset to include multiple coal mines or similar excavation sites with different geological conditions and blasting parameters. This broader dataset would allow for a more comprehensive evaluation of model generalizability. Next, to suit most cases, an extensive database about the geological mechanics parameters should be established to suit most cases.

Arithmetic Optimization Algorithm (AOA) is adopted to optimize the hyperparameters and shows effective performance in predicting PPV. However, future studies could focus on exploring other optimization algorithms, which could yield even better hyper-parameter tuning results for ML models.

## Conclusion

This study demonstrates how empirical equations and machine learning (ML) algorithms can be systematically utilized for predicting ground vibration. This work’s contributions can be divided into four aspects: the empirical equation improvement, the calculation of correlations between the PPV and other input parameters, selection of ML algorithms, and feature contributions in ML model. Based on the analysis results, the following conclusions can be drawn:

1. The study presented an overview of the common empirical equations and the influence of burden on blast-induced ground vibration. The summerision showed that the impact of burden on ground vibration is controversial.
2. An empirical equation that considers burden (Eq. 7) using Buckingham’s Pi Theorem is established. Equation (7) achieves better PPV prediction performance than the majority of other empirical equations. Therefore, Eq. (7) can be used to predict PPV when the burden is sonsidered as the one of major parameters.
3. The Pearson correlation coefficient between *R* and *PPV* is − 0.67, whereas the correlation coefficients between the other three parameters (*Q*, *HD*, and *B*) and *PPV* are − 0.15, − 0.28, and − 0.31, respectively. These results indicate that the three parameters’ influence on predicting *PPV* is less significant than that of *R*.
4. The prediction performance of six commonly used ML algorithms (namely, RR, LR, SVR, ANN, KNN, and RF) was studied in detail. As a result, KNN and RF were selected as the best-performing models and were further optimized in the next training round. The results of RR, LR, ANN, and SVR show that these algorithms are not suitable for predicting PPV in the studied situation. In particular, the results demonstrate the infeasibility of utilizing linear algorithms (e.g., RR and LR) to predict PPV.
5. RF and KNN models were developed following the outlined methodology. The AOA alogrithm was employed to detect the optimal hyper-parameters and judge whether the models’ accuracy can be improved. The performance assessment revealed that AOA-RF outperformed AOA-KNN (regarding performance assessments, REC(values of AOC) and UA) after the second training round. Overall, RF is suggested as the optimal algorithm for predicting PPV in scenarios similar to the study reported herein. SHAP was adopted to explan the parameter contribution to the AOA-RF, *R* is a key factor that significantly contributes to the model. However, Burden have a lesser influence in comparison.

## Data Availability

Data sets generated during the current study are available from the corresponding author on reasonable request. The PPV data are available but restrictions apply to the availability of these data, which were used under license for the current study, and so are not publicly available.

## Abbreviations

PPV: Peak particle velocity
ML: Machine learning
R: Distance from blasting center to monitored point
HD: Hole depth
*B*: Burden
*S*: Spacing
*Q*: Maximum charge weight per delay
CL: Charge Length
HR: Hole diameter
*E*: Young’s modulus
BI: Blastability index
St: Stemming
PF: Power factor
UCS: Uniaxial compressive strength
N: Rows per blast
Pv: P-wave velocity
Sv: S-wave velocity
H: Bench height
VoD: Velocity of detonation of explosive
DoE: Density of explosive
PR: Poisson’s ratio
Δt: Delay time
HN: Hole number
*K*: Empirical constants
$$\alpha$$: Empirical constants
$$\beta$$: Empirical constants
*f*_*R*_: Rock hardness
$\rho$: Rock mass density
*c*: Phase velocity
$$\pi_{{\text{n}}}$$: Dependent variables
RR: Ridge regression
LR: Lasso regression
ANN: Artificial neural network
SVR: Support vector regression
CART: Classification and regression tree
ANFIS: Adaptive neuro-fuzzy inference system
GEP: Gene expression programming
KNN: K-Nearest Neighbors
${\text{Cov}}\left( {x,y} \right)$: Population covariance matrix
$\sigma_{x}$, $\sigma_{{\text{y}}}$: Deviations of *x* and* y*
$$E\left( x \right)$$: Average *x* values
$$E\left( {\text{y}} \right)$$: Average *y* values
$$x_{{\text{i}}}$$,$$y_{{\text{i}}}$$: Variable values
$\theta$: Vector of weight coefficients
*w*: Weighting matrix
*b*: Bias term
*γ*: Maximum margin
*C*: Regularization parameter
$$\xi_{k} ,\xi_{k}^{*}$$: Slack variables
$f$: Activation function or the action function
${\text{n}}_{tree}$: Number of decision trees
$$X_{UB}$$: Upper boundaries
$$X_{{{\text{L}}B}}$$: Lower boundaries
$$C_{iter}$$: Current iteration
$$M_{iter}$$: Iterations
$$\mu$$: Control variable
$\alpha_{{\text{A}}}$: Parameter that defines the accuracy
$${\text{x}}_{m}$$: Measured value
$${\text{x}}_{{\text{p}}}$$: Predicted value
${\text{x}}_{{{\text{mean}}}}$: Mean of measured value
R^2^: Coefficient of determination
MSE: Mean square error
RSME: Root means square error
VAF: Variance account for
MAE: Mean absolute error
NMBE: Mean bias error
MAPE: Mean absolute percentage error
NS: Nash–Sutcliffe Efficiency
IOA: Index of agreement
IOS: Index of scatter
a20: A20-index
PI: Performance index
REC: Regression error characteristics
AOC: Area over this curve
UA: Uncertainty analysis
MOE: Margin of Error
SD_ev_: Standard deviation
SE: Standard error
ME: Margin of Error at a 95% confidence level
WBC: White blood cell count
UB: Upper bound
LB: Lower bound
SHAP: Shapley additive explanations
